# Action potential wavelength restitution predicts alternans and arrhythmia in murine *Scn5a*^+/−^ hearts

**DOI:** 10.1113/jphysiol.2013.254938

**Published:** 2013-07-08

**Authors:** Gareth D K Matthews, Laila Guzadhur, Ian N Sabir, Andrew A Grace, Christopher L-H Huang

**Affiliations:** 1 Physiological Laboratory, University of Cambridge Downing Street, Cambridge CB2 3EG, UK; 2 Department of Biochemistry, Hopkins Building, University of Cambridge Cambridge CB2 1QW, UK; 3 School of Clinical Medicine, University of Cambridge, Addenbrookes Hospital Cambridge CB2 0QQ, UK; 4 Rayne Institute, St Thomas’ Hospital Westminster Bridge Road, London SE1 7EH, UK

## Abstract

Reductions in cardiac action potential wavelength, and the consequent wavebreak, have been implicated in arrhythmogenesis. Tachyarrhythmias are more common in the Brugada syndrome, particularly following pharmacological challenge, previously modelled using *Scn5a*^+/−^ murine hearts. Propagation latencies and action potential durations (APDs) from monophasic action potential recordings were used to assess wavelength changes with heart rate in Langendorff-perfused wild-type (WT) and *Scn5a*^+/−^ hearts. Recordings were obtained from right (RV) and left (LV) ventricular, epicardial and endocardial surfaces during incremental pacing, before and following flecainide or quinidine challenge. Conduction velocities (θ′), action potential wavelengths (λ′= APD ×θ′), and their corresponding alternans depended non-linearly upon diastolic interval (DI). Maximum θ′ was lower in *Scn5a^+/−^* RV epicardium than endocardium. Flecainide further reduced θ′, accentuating this RV conduction block. Quinidine reduced maximum θ′ in WT and caused earlier conduction failure in the RV of both *Scn5a^+/−^* and WT. Use of recovery wavelengths (λ′_0_= DI ×θ′) rather than DI, provided novel λ restitution plots of λ′ against λ′_0_, which sum to a basic cycle distance permitting feedback analysis. λ′ restitution gradient better correlated with alternans magnitude than either APD or θ restitution gradient. The large differences in θ′ and APD restitution contrasted with minor differences in maximum λ′ between epi- and endocardia of untreated hearts, and quinidine-treated WT hearts. Strikingly, *all* regions and conditions converged to a common instability point, implying a conserved relationship. Flecainide or quinidine decreased the pacing rates at which this occurred, through reducing basic cycle distance, in the *Scn5a^+/−^* RV epicardium, directly predictive of its arrhythmic phenotype.

**Key points**

- Mice which are haploinsufficient in the *Scn5a*^+/−^ gene have reduced cardiac sodium channel (Na_v_1.5) density and are used to model the Brugada syndrome.
- Conduction velocity restitution showed lower initial values and earlier points of failure during incremental pacing in the murine *Scn5a*^+/−^ right ventricle (RV) epicardium particularly when treated with flecainide.
- The broadness of the conduction velocity restitution function was a poor indicator of arrhythmia or alternans. Conduction velocity alternans occurred abruptly and was more marked in the flecainide-treated *Scn5a*^+/−^ RV epicardium.
- Introduction of wavelength restitution yielded functions that converged to a common instability condition in contrast to action potential duration (APD) or conduction velocity restitution.
- This occurred at significantly lower heart rates in *Scn5a*^+/−^ RV epicardium following flecainide or quinidine challenge, corresponding to a smaller total wavelength (basic cycle distance) resulting from a reduction in conduction velocity.
- Wavelength restitution was superior at predicting alternans than either APD or conduction velocity restitution.

## Introduction

Ventricular arrhythmia, particularly ventricular fibrillation (VF), represents a major cause of death worldwide (Chugh *et al.* 2008). Risk of ventricular arrhythmia may be inherited, as in the Brugada syndrome (BrS). Previous work suggested that the mechanism of VF initiation involves breakdown of the propagating action potential (AP) wavefront into multiple wavelets, which cause scroll wave formation (Davidenko *et al.* 1995; Zaitsev *et al.* 2000). This leads, in a positive feedback manner, to further wavebreaks, the wavelets of which follow the chaotic and meandering conduction pathways that characterise VF. Wavebreak is proposed to result from heterogeneities existing within the cardiac tissue (Weiss *et al.* 2005). These may either be anatomical such as an ischaemic area or vascular structure, or they may be functional such as an increased dispersion of refractoriness resulting in pockets of inactivatable tissue.

The travelling wave has an associated excitation wavelength (λ) given by the product of its action potential duration (APD) and conduction velocity (θ). The longer the λ, the less likely that areas of depolarisation and repolarisation will meet upon encountering tissue heterogeneity (Fig. 1*C*). There is normally a sufficient safety factor for the wave to completely pass over the heterogeneity, but decreases in λ below the extents of the heterogeneities increase the probability of wavebreak (Fig. 1*D*). Rapid heart rates decrease both APD and θ, as characterised by their respective restitution functions.

**Figure 1:**
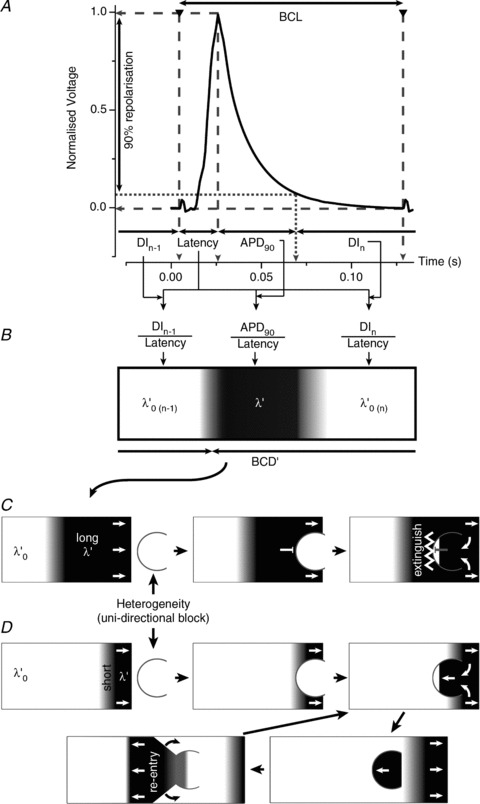
Summary of wavelength calculations from primary electrophysiological data and their relationship to the concept of wavebreak re-entry *A*, typical monophasic action potential obtained from the *Scn5a^+/−^* RV epicardium upon which is superimposed indications of BCL, APD90, latency and DI of both the current (*n*^th^) and its preceding ((*n*–1)^th^) action potential. These form the basis for computations of the wavelength parameters representing λ′ and λ′_0_ (*B*). These sum together to give the BCD′ which is primarily determined by the pacing rate and conduction velocity of the tissue. *C*, long wavelength AP encountering a heterogeneity which may cause a unidirectional conduction block. This may be functional; due to wavelength differences, discordant alternans leading to nodal line formation or refractoriness, or anatomical due to anisotropy or damage. As the long wavelength AP passes over the heterogeneity, retrograde propagation is blocked by the back of the propagating wave, causing it to be extinguished, leaving only the orthograde excitation wave to continue. *D*, short wavelength AP encountering the same-sized heterogeneity. In this case the waveback has passed the heterogeneity before the retrograde excitation has passed through the unidirectional block. Therefore a new propagating retrograde wave is set up which may in turn back-excite the orthograde direction setting up a sustained re-entrant circuit.

APD restitution, where APD is plotted against diastolic interval (DI), has previously been extensively studied for arrhythmia prediction (Nolasco & Dahlen, 1968). This elegant method incorporates an iterative feedback loop generating unstable APD alternans from a critical point (DI_crit_) in the pacing regime where the function gradient reaches one. Alternans, where APDs alternate between long and short lengths, is thought to be a major mechanism for generating an arrhythmic substrate. Discordant alternans, where APD in adjacent areas of cardiac tissue alternate out of phase (one area has a short APD and the other a long APD), result in an APD gradient across that area of myocardium with an intermediate region, known as the nodal line, at which no alternans occurs (Pastore *et al.* 1999). If triggered activity occurs within the short-APD area, it propagates radially towards the nodal line. Pathways which take the most direct route meet the nodal line when the long-APD area is still depolarised and therefore refractory, causing their extinguishment. However, propagation via less direct, longer distance pathways reaches the nodal line later when the long-APD area has repolarised (see Figs. 1 and 2 of Weiss *et al.* 2006). This allows the triggered AP to re-excite the repolarised area, causing the generation of a re-entrant circuit (Qu *et al.* 2000; Watanabe *et al.* 2001). Re-entry paces the cardiac tissue at a high rate, ventricular tachycardia (VT), increasing the likelihood of wavebreak and degeneration to VF. However, whilst APD restitution has been successful in some studies at predicting arrhythmogenesis (Narayan *et al.* 2007*b*), others do not confirm a clear-cut relationship (Dorenkamp *et al.* 2013).

**Figure 2:**
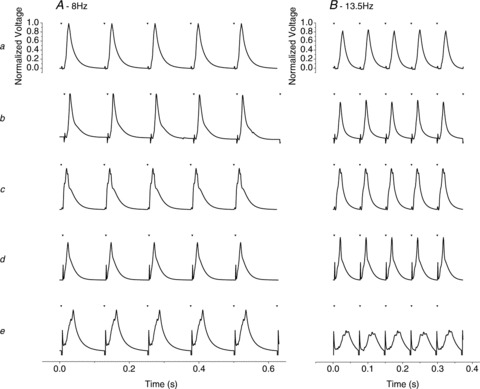
MAP recordings obtained during the incremental pacing protocol Typical traces observed at resting (8 Hz; *A*) and exercising (13.5 Hz, *B*) heart rates in the WT RV epicardium (*a*), WT RV endocardium (*b*), flecainide-treated WT RV epicardium (*c*), *Scn5a*^+/−^ RV epicardium (*d*) and flecainide-treated *Scn5a*^+/−^ RV epicardium (*e*). The flecainide-treated *Scn5a*^+/−^ RV epicardium shows longer latencies at both pacing rates and alternans at the higher rate.

Na^+^ channel haploinsufficient, *Scn5a^+/−^* mice have been used to model BrS, in which *SCN5A* mutations occur in 30% of cases (Alings & Wilde, 1999). *Scn5a^+/−^* hearts replicate many features of the human condition. They show VT at low heart rates and more VF following flecainide challenge at higher heart rates (Matthews *et al.* 2012). They have similar ECG features including ST-segment elevation in the right precordial leads (Martin *et al.* 2010). *Scn5a^+/−^* hearts further demonstrate fibrotic changes with ageing in common with the human condition (Jeevaratnam *et al.* 2011). They also have an increased propensity to APD alternans (Matthews *et al.* 2010) paralleling recent reports in humans (Kofune *et al.* 2009; Liu & Chang, 2013). This may reflect their reduced Na^+^ channel expression (Martin *et al.* 2012*b*) leading to disturbances in θ, APD and refractoriness. These factors will result in disturbances to the following AP, causing alternans.

Recent comparisons of directly measured APD alternans and its relationship to the restitution function gradient in the *Scn5a*^+/−^ mouse demonstrated that both APD alternans and restitution properties were abnormal in the right ventricular (RV) epicardium following flecainide challenge, supporting APD restitution theory. However, the exact relationship between the two variables was non-linear and continuous, rather than linear with an unstable threshold. This deviation from the predicted relationship resulted in an underestimation of the magnitude of alternans, suggesting that the APD restitution theory is incomplete (Matthews *et al.* 2012). Factors other than voltage feedback such as intracellular Ca^2+^ homeostasis (Chudin *et al.* 1999) and θ (Banville & Gray, 2002) may therefore be generating alternans. Displays of θ using a restitution function use parameters with differing dimensions of velocity and time, and thus fail to incorporate a feedback relationship leading to instability. Nevertheless, recent multielectrode array studies using *Scn5a*^+/−^ mice have demonstrated cardiac conduction defects (Stein *et al.* 2011).

The present study utilises the haploinsufficient *Scn5a^+/−^* mouse to develop a unified criterion, in terms of λ, for feedback instabilities leading to ventricular arrhythmogenesis. The study first demonstrates abnormal θ restitution and alternans properties that might generate arrhythmic substrate. It then incorporates these findings into a novel λ restitution analysis as described in the Theory section. The advantage of this method over previous attempts to measure the adaptation of λ with rate (Weber, 2011; Lou *et al.* 2012) is that it incorporates feedback criteria and can thus predict instability in the form of alternans.

## Methods

### Ethical approval

Experiments were approved by the University of Cambridge ethics review board under a UK project licence for studies of cardiac arrhythmia. Procedures were performed in regulated premises and approved under the UK Animals (Scientific Procedures) Act (1986). Mice were maintained in plastic cages at 21 ± 1°C, subject to 12 h day^-1^ light–dark cycles, and had free access to water, sterile chow (RM3 Maintenance Diet, SDS, Witham, UK), bedding and environmental stimuli. Mice were killed (Schedule 1) by cervical dislocation. All procedures were rapid, humane and minimised stress to the animal. No recovery, anaesthetic or surgical procedures were required. Thirty-nine WT mice were used, three of which were excluded. Of these 16 were male and 23 were female. Twenty-nine *Scn5a^+/−^* mice were used, one of which was excluded. Of these 15 were male and 14 were female.

### Experimental studies

The experimental procedures have previously been described in detail (Matthews *et al.* 2012). Male and female, WT and *Scn5a*^+/−^ 129/sv mice (Papadatos *et al.* 2002; Harlan, UK) aged 3–5 months were used to investigate electrophysiological properties without the structural changes associated with ageing in the male (Jeevaratnam *et al.* 2011). Hearts obtained following bilateral sternectomy were cannulated and perfused with filtered Krebs–Henseleit buffer (NaCl (119 mm), NaHCO_3_ (25 mm), KCl (4 mm), MgCl_2_ (1 mm), KH_2_PO_4_ (1.2 mm), CaCl_2_ (1.8 mm), glucose (10 mm) and sodium pyruvate (1.8 mm) bubbled with 95% O_2_/5% CO_2_; British Oxygen Company, Manchester, UK) to achieve a pH of 7.4, at a constant flow rate of 2.5 ml min^−1^.

Epicardial monophasic action potentials (MAPs) were recorded by placing a MAP electrode (Hugo Sachs, Harvard Apparatus, UK) upon the left (LV) and right ventricular epicardial surface with the heart apposed to a plastic mechanical support. Endocardial MAP recordings were taken using an electrode constructed by intertwining two pieces of Teflon-coated silver wire, whose ends were stripped for 1 mm, galvanically chlorided and splayed. LV or RV electrode positioning was verified at the end of each experiment by methylene blue staining. MAP recordings were obtained, amplified (Neurolog NL100 preamplifier; NL104 amplifier, Digitimer, Welwyn Garden City, UK), band-pass filtered (NL125/126 filter; 0.5 Hz to 1.0 kHz) and sampled at 5 kHz (micro1401 interface) for display using Spike II software (Cambridge Electronic Design, Cambridge, UK). Hearts were paced at twice threshold voltage using a bipolar platinum-coated stimulating electrode placed on the ventricular septum, connected to a DS2A-Mk.II stimulator (Digitimer) controlled using Spike II. Initial stimulation using constant 8 Hz pacing was used to find MAP recordings with stable baselines, triangular waveforms, rapid upstroke phases and smooth recoveries to baseline (Knollmann *et al.* 2001). The incremental pacing protocol was then imposed beginning at a 134 ms basic cycle length (BCL) for 100 stimulations, followed by a decrement of 5 ms. This was repeated until hearts showed either entry into 2:1 block or arrhythmogenesis. Following recording, the preparation was checked for the continued presence of intrinsic activity, healthy colouration and capacity to be paced at 8 Hz. Arrhythmia frequently cardioverted to sinus rhythm following 8 Hz pacing. Hearts remaining in arrhythmia always returned to sinus rhythm and re-established viable MAP appearances (Knollmann *et al.* 2001) after application of cold buffer followed by 5 min of 8 Hz pacing.

Both male and female *Scn5a^+/−^* mice showed VF and VT. Flecainide or quinidine were added to give concentrations of 10 μm and 5 μm, respectively, as used in previous work (Belhassen *et al.* 2004; Stokoe *et al.* 2007; Matthews *et al.* 2010), and hearts perfused with test agent for 4 min before study. Combinations or sequential additions of these agents were not investigated due their lipophilicity preventing complete washout.

### Construction and analysis of conduction velocity and λ restitution curves

Latencies, reciprocal latencies (θ′) (Fig. 1*A*), and thus the corresponding active (λ′) and resting (λ′_0_) wavelengths (Fig. 1*B*), defined in the Theory section, were calculated on an AP-by-AP basis for each heart. This involved automatic detection of the time to peak following stimulation as well as 90% repolarization to baseline (APD_90_). The corresponding DI was calculated from the BCL and APD_90_ using the relationship DI = BCL – APD_90_ (Fig. 1*A*). This method permitted an accurate and consistent measure of APD and θ, which did not rely on measurement of upstroke gradient, as this is not necessarily fully reproduced by MAP recordings. Data were plotted and processed for individual hearts in each group. Results from hearts whose parameterisations were closest to the group medians were chosen for display rather than averaging over the entire group. Averaging results in inappropriate smoothing of the original curved data due to the constituent functions being slightly shifted. Curves of θ′ against DI, λ′ against DI and λ′ against λ′_0_ were plotted using OriginPro8 (OriginLab Corporation, Northampton, MA, USA) and approximated by a simple, mono-exponential decaying growth function (Pak *et al.* 2004):


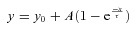
(1)

Here, *y* represents either θ′ or λ′, *x* represents DI or λ′_0_, and *y*_0_, *A* and τ are constants of the mono-exponential function obtained by a least squares fitting equation applied as follows:


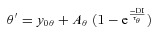
(2)


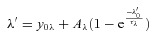
(3)

The gradient (*m*) of eqn (1) is then given by:


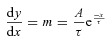
(4)

This assumes its maximum value (*m*_max_) at the DI or λ′_0_ at the effective refractory period (ERP):


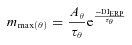
(5)


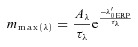
(6)

The critical λ′_0_ (λ′_0crit_) is the λ′_0_ at which *m*_λ_ of the λ restitution curve equals one and is calculated by:


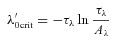
(7)

The corresponding BCL_crit(λ)_ was obtained by finding the value of BCL at λ′_0crit_. Furthermore, the DI_limit(θ)_ or the λ′_0limit_ are the *x*-axis intercepts of the mono-exponential functions. They occur when θ′ or λ′ collapse to zero and are calculated by:


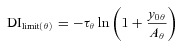
(8)


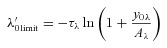
(9)

The maximum possible value of either θ′ (θ′_max_) or λ′ (λ′_max_) was calculated by the equations:


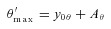
(10)


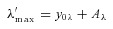
(11)

### Detection and quantification of alternans

An established alternans detection algorithm (Matthews *et al.* 2010) identified sequences of ≥10 APs showing alternans on an AP-by-AP basis. Alternans magnitude was determined from absolute differences in parameters between successive APs (Matthews *et al.* 2012) for calculation of the mean and standard error of the mean (SEM) during each 100 stimulation run at every BCL. The magnitude of alternans was plotted against DI or λ′_0_ (OriginPro8: OriginLab Corporation). The progression of the λ′ alternans could be described by an exponential decay function of the form:


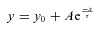
(12)

where *y* represents alternans magnitude, *x* represents λ′_0_, and *A*, τ and *y*_0_ are fitting constants. The constant τ represents the decay in *y* with increasing *x* to 1/e of its maximum value at *x*= 0 giving an index of the broadness of the function:


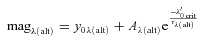
(13)

However, the progression of θ′ alternans did not fit a simple mono-exponential function as alternans occurred abruptly. Thus, its characterisation compared values of θ′ alternans at specific pacing rates. The gradient of eqn (12) is given by:


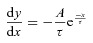
(14)

This assumes a maximum value (*m*_maxλ(alt)_) at the λ′_0_ value observed at the BCL prior to reaching the effective refractory period λ′_0ERP_:


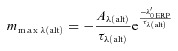
(15)

A critical λ′_0_ value on the alternans curve at a gradient of –1, reflecting the critical λ′_0_ value on the restitution curve where the gradient is equal to 1 is given by:


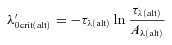
(16)

The BCL_λ(alt)_ was calculated as the BCL at the λ′_0crit(alt)_.

The λ′_0_ at the point of intersection (λ′_0χ_) between the magnitude of alternans and the restitution curves describes the situation predicting 2:1 block due to alternans. λ′_0χ_ was obtained by equating the right-hand sides of eqns (3) and (13), then solving the resulting function by Newton's method. The BCL_λ(χ)_ is the BCL at the λ′_0χ_.

### Relationship between the magnitude of λ restitution gradient and alternans

The gradient of the λ restitution function is hypothesised to predict the magnitude of the corresponding alternans, mag_λ(alt)_. This was explored by equating the exponential functions shown in eqns (4) and (13), permitting mag_λ(alt)_ to be plotted against *m*_λ_ through the following analytical solution:


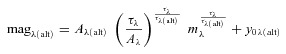
(17)

The function describes a smooth dependence of the form:


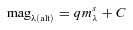
(18)

where *q*, *s* and *C* represent a coefficient, power and intercept, respectively. This resulting function was compared to a line of equality, mag_λ(alt)_=*m*_λ_, to assess its adherence to a direct relationship.

### Statistical testing and data display

Primary electrophysiological data derived from the MAP traces were normally distributed, and were plotted as means and SEMs on the restitution graphs. However, data derived from fitting to the non-linear functions were not normally distributed (Shapiro–Wilk test <0.05). Such data were accordingly expressed as medians (Mdn) and interquartile ranges (IQRs), written in the form Mdn(IQR).

Statistical testing (SPSS, IBM, Somers, USA) for significant differences between different experimental groups used non-parametric methods. Kruskal–Wallis tests, which yielded values of chi-squared (χ^2^), degrees of freedom (d.f.) and *P* value (*P*), were applied in groups consisting of the entire data set, the WT and *Scn5a*^+/−^ hearts, in the presence and absence of flecainide or quinidine, at LV or RV, epicardial or endocardial recording sites. These prompted pairwise analysis using Mann–Whitney *U* tests, whose results are displayed as their *U*-statistic (*U*), *P* value (*P*) and effect size (*r*). Effect size was calculated as *r*=*Z*/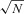, where *Z* is the value of the *Z*-statistic and *N* is the number of hearts studied. Comparisons between genotypes, regions and drugs were unpaired as they involved comparing separate *Scn5a*^+/−^ and WT hearts. Furthermore, within any given group, it was not possible to obtain endocardial and epicardial recordings in every chamber in the same heart, due to the procedures required for endocardial recording. Recordings were made in sequence at constant distances from the stimulation. Hearts were exposed to either flecainide or quinidine, and thus different hearts were used in control and test data sets. For each genotype, pairwise statistical tests compared the results of varying the RV or LV, epicardial or endocardial recording site under each pharmacological condition. They further compared the results obtained at each site, for any given genotype, with and without flecainide or quinidine treatment. They also compared findings between WT and *Scn5a*^+/−^ at any recording site for each pharmacological condition. Finally, they compared the actions of flecainide against quinidine at each site for each genotype. All significance thresholds were recalculated using an Bonferroni–Holm correction to *P*≤ 0.05 (Jaccard & Wan, 1996). All *P* values displayed have been so adjusted, correcting for type 1 error, whilst minimising type 2 error.

Significant values are displayed in the text along with the appropriate statistical test. Significant Kruskal–Wallis tests are displayed in Supplemental data section 6 (available online only). Mann–Whitney *U* tests for τ_λ,_τ_λ(alt)_ *m*_max(λ),_ as well as some BCD and latency values are included in Supplemental data section 7 for completeness. All parameters, including fitting constants and derived variables, are tabulated in Supplemental data (Tables 1–5). Figures for the RV are displayed in the results, whereas non-significant LV figures are displayed in the Supplementary Figures 1–6.

### Glossary of symbols

*A*, amplitude fitting constant of the monoexponential function, subscript θ denotes conduction velocity restitution, subscript λ denotes wavelength restitution and subscript λ(alt) denotes wavelength alternans plots; BCL, basic cycle length; BCL_crit(λ)_, the BCL at which λ′_0crit_ occurs; BCL_critλ(alt)_, the BCL at which λ′_0crit(alt)_ occurs; BCL_λ(χ)_, the BCL at which λ′_0χ_ occurs; *C*, offset of the analytical function comparing restitution and alternans which is equal to *y*_0λ(alt)_; DI_crit_, critical diastolic interval where gradient of the APD restitution curve is unity; DI_limit(θ)_, the predicted diastolic interval where conduction velocity would equal zero regardless of stimulus voltage; DI_r_, residual DI between repolarisation and next stimulation; mag_λ(alt)_, magnitude of λ′ alternans; *m*, function gradient, subscript θ denotes conduction velocity restitution and subscript λ denotes wavelength restitution; *m*_max_, maximum gradient; *m*_max(λ)_, maximum gradient of the λ restitution curve; *m*_maxλ(alt)_, maximum gradient of the λ alternans against λ′_0_ curve; *N*, number of hearts; *q*, coefficient of the analytical function comparing restitution and alternans; *r*, effect size of Mann–Whitney *U* test; *s*, power of the analytical function comparing restitution and alternans; U, U-statistic of the Mann-Whitney test; *y*_0_, offset fitting constant of the monoexponential function, subscript θ denotes conduction velocity restitution, subscript λ denotes wavelength restitution and subscript λ(alt) denotes wavelength alternans plots; α, initial estimate used for Newton's Method; θ, conduction velocity; θ′, reciprocal latency which is proportional to conduction velocity if distance travelled remains constant; θ′_max_, maximum conduction velocity predicted by restitution curve; λ, excited wavelength, ′ indicates calculation without distance factor; λ_0_, resting wavelength, ′ indicates calculation without distance factor; λ′_0crit_, the λ′_0_ value at which the λ restitution curve is at unity; λ′_0ERP_, the value of resting wavelength at the effective refractory period; λ′_0limit_, the value of resting wavelength at which the excited wavelength is zero regardless of stimulus voltage; λ′_max_, maximum wavelength predicted by restitution curve; λ′_0crit(alt)_, λ′_0_ corresponding to negative unity gradient in the λ restitution curve; λ′_0χ_, the resting wavelength at the intersection between restitution and alternans plots; τ, time constant fitting constant of the monoexponential function, subscript θ denotes conduction velocity restitution, subscript λ denotes wavelength restitution and subscript λ(alt) denotes wavelength alternans plots; χ^2^, chi-squared value of Kruskal–Wallis test.

### Theory section

A novel restitution analysis involving wavelengths of excited and recovering regions of the myocardium extended the previously established APD restitution theory. The latter relates APD and DI through a feedback mechanism where the APD is determined by the preceding duration spent at the resting potential. These variables were interconnected by the equation:


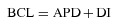
(19)

The DI consists of two major components: the time spent at the resting potential following repolarisation before the subsequent AP (DI_r_), and the latency between the imposition of the subsequent stimulus and the upstroke of the following AP. Hence the above equation may be written more completely as:


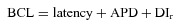
(20)

The distance, λ, may be expressed as the product of velocity and duration. In the present context:


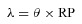
(21)

Refractory period (RP) is difficult to measure at any given rate and varies with stimulus voltage. However, it depends upon the APD giving the approximation:


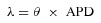
(22)

θ is a measure of the distance of tissue through which the AP has propagated in a given time. Thus:


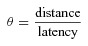
(23)

Equation (22) is converted into wavelength dimensions by multiplying by θ giving:


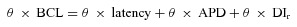
(24)

yielding the novel restitution relationship:


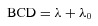
(25)

The basic cycle distance (BCD) equals the product of the BCL and θ. λ is the excited wavelength given by the product of θ and APD. λ_0_ is the resting wavelength given by the product of θ and (DI_r_+ latency) which equals the product of θ and DI. Equation (25) is thus similar in form to eqn (19) and allows plotting of iterative restitution functions with a critical instability value at unity gradient.

Experiments maintained the interelectrode distance between the stimulating and recording electrode at 5 mm. However, it is impossible to calculate the actual conduction pathway with any available technique, as even a multielectrode array does not include a *z*-axis recording. For this reason we approximate conduction velocity using:


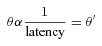
(26)

Thus:


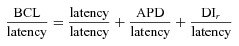
(27)

This gives a similar relationship to eqn (25):


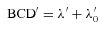
(28)

where the prime (′) shows that distance has not been incorporated. The above makes the assumption that keeping the interelectrode distance and stimulation voltage to threshold ratio constant results in a constant conduction pathway. This assumption is reasonable when conduction velocity curves give monoexponential declines as observed (Derksen *et al.* 2003), suggesting propagation through preferred pathways of excitation due to Purkinje fibre innervation.

## Results

MAPs were compared in RV and LV, epicardia and endocardia, of WT and *Scn5a*^+/−^ ventricles, before and following addition of either flecainide or quinidine. Figure 2 illustrates typical recordings from WT (*a*, *b*, *c*) and *Scn5a^+/−^* (*d* and *e*) RV before and following flecainide at resting (8 Hz, *A*) and exercising heart rates (13.5 Hz, *B*). Latencies were clearly increased at the higher pacing rate and following addition of flecainide. In the absence of pharmacological agent, latency alternans only appeared at the end of the pacing protocols. However, such effects became marked at longer BCLs and took place with larger magnitudes in flecainide-treated *Scn5a*^+/−^ RV epicardium. Similar effects occurred in quinidine-treated WT RV endocardium.

### Measures of conduction latency

Conduction velocities were assessed from AP latencies at the BCL just preceding refractoriness. Before pharmacological intervention, these latencies were consistently greater in *Scn5a*^+/−^ RV epicardium than in either the *Scn5a*^+/−^ RV endocardium or WT RV epicardium (24.59(6.68) ms *vs*. 16.74(9.20), or 18.31(6.99) ms; *U*= 40.0, *P*= 0.005, *r*= 0.53 and *U*= 158.0, *P*= 0.002, *r*= 0.47, respectively). This implies a right-sided conduction block within the *Scn5a^+/−^*, recapitulating the right bundle branch block seen in BrS. Either flecainide or quinidine slowed conduction velocity in both genotypes as expected from their Na^+^ channel blocking activity. Flecainide increased the latencies in *Scn5a*^+/−^ RV epicardium (from 24.59(6.68) to 38.20(18.94) ms; *U*= 21.0, *P*= 1.6 × 10^−4^, *r*= 0.67). This additionally resulted in an epicardial–endocardial difference in the *Scn5a*^+/−^ RV (38.20(18.94) *vs*. 25.81(19.75) ms; *U*= 20.0, *P*= 0.05, *r*= 0.50). Quinidine increased conduction times in all WT recording sites, particularly in the RV endocardium (from 19.01(16.46) to 41.41(12.89) ms; *U*= 16.0, *P*= 0.018, *r*= 0.57). Quinidine increased *Scn5a^+/−^* RV epicardium conduction latencies (from 24.59(6.68) to 31.88(12.02) ms; *U*= 72.0, *P*= 0.041, *r*= 0.40).

### Conduction velocity restitution curves

Figure 3 exemplifies typical restitution plots of θ′ against DI through all the explored regions and conditions. Previous studies (Banville & Gray, 2002; Qu *et al.* 2006) had qualitatively described these as being either broad or narrow, relating this to high or low arrhythmic risk, respectively.

**Figure 3:**
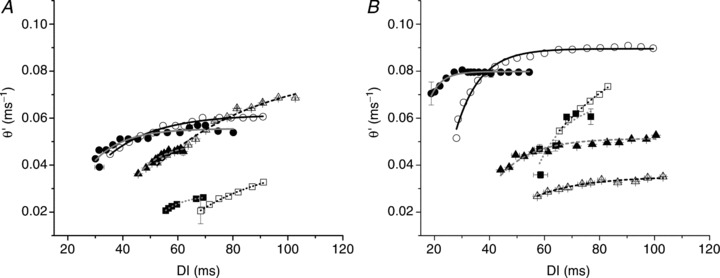
Conduction velocity restitution curves Plots of θ′ against DI obtained from typical (*N* = 1) WT (open symbols; black lines) and *Scn5a*^+/−^ (filled symbols; grey lines) hearts in the RV epicardium (*A*) and RV endocardium (*B*), before (circles; continuous line) and following addition of flecainide (squares; dashed line) or quinidine (triangles; dotted line). The data displayed represent hearts showing the Mdn value of θ′_max_ in the experimental group concerned. Both the vertical position and shape of such curves could vary greatly between groups. Flecainide and quinidine both typically reduced conduction velocity, but this was marked in the flecainide-treated *Scn5a^+/−^* RV epicardium.

Variations in function width were characterised by a time constant (τ_θ_). τ_θ_ was narrower in the WT LV endocardium than in the corresponding epicardium (6.91(5.22) *vs*. 16.12(29.18) ms; *U*= 20.0, *P*= 0.021, *r*= 0.56). However, τ_θ_ was broadened in the WT LV endocardium by either flecainide or quinidine (from 6.91(5.22) ms to 27.77(26.43) ms; *U*= 10.0, *P*= 0.049, *r*= 0.61, and to 40.1(34.81) ms; *U*= 4.0, *P*= 0.039, *r*= 0.69, respectively). In the presence of flecainide, τ_θ_ was broader in the WT LV epicardium than the corresponding *Scn5a*^+/−^ (72.67(50.90) *vs*. 8.13(4.84) ms; *U*= 3.0, *P*= 0.041, *r*= 0.69). These findings are not consistent with the RV arrhythmic phenotype seen in the *Scn5a^+/−^* mouse (Matthews *et al.* 2012), limiting the use of function broadness for arrhythmic risk stratification.

θ′ restitution functions also provided a maximum value of θ′ at the lowest heart rates (θ′_max_). θ′_max_ was similar in WT RV epicardium and endocardium (0.07(0.03) *vs*. 0.08(0.02) ms^−1^; *U*= 54.0, *P*= 0.129). In contrast θ′_max_ was lower in *Scn5a^+/−^* RV epicardium than the corresponding endocardium (0.05(0.01) ms^−1^ *vs*. 0.08(0.03) ms^−1^; *U*= 27.0, *P*= 0.05, *r*= 0.57). Flecainide further reduced θ′_max_ in the *Scn5a*^+/−^ RV epicardium (from 0.05(0.01) to 0.03(0.028) ms^−1^; *U*= 18.0, *P*= 0.026, *r*= 0.54). Quinidine reduced θ′_max_ in the WT RV endocardium and WT LV epicardium (from 0.08(0.02) to 0.04(0.02) ms^−1^; *U*= 4.0, *P*= 0.014, *r*= 0.71 and from 0.10(0.06) to 0.04(0.04) ms^−1^; *U*= 19.0, *P*= 0.030, *r*= 0.52). Quinidine resulted in a θ′_max_ difference between the WT RV epicardium and endocardium (0.08(0.03) *vs.* 0.04(0.02) ms^−1^; *U*= 1.0, *P*= 0.017, *r*= 0.79). In the presence of quinidine θ′_max_ was smaller in the *Scn5a*^+/−^ RV epicardium than the corresponding WT (0.05(0.020) *vs*. 0.08(0.0299) ms^−1^; *U*= 5.0, *P*= 0.014, *r*= 0.71). These findings demonstrate the RV conduction block in the *Scn5a*^+/−^ and its exacerbation by flecainide persists at very low heart rates such as those occurring during sleep or sinus node dysfunction. Quinidine's actions predominantly targeted the WT, but also abolished the θ′_max_ difference in the *Scn5a^+/−^* RV, which may therapeutically normalise the dispersion in arrival times.

The limiting DI value at which conduction velocity fell to zero (DI_limit(θ)_) at high heart rates was increased by flecainide in *Scn5a*^+/−^ RV epicardium and corresponding endocardium (from 16.57(17.89) to 44.66(22.16) ms; *U*= 9.0, *P*= 0.004, *r*= 0.65, and from 14.55(11.75) to 40.10(18.99) ms; *U*= 1.0, *P*= 0.005, *r*= 0.81). Quinidine produced smaller increases in DI_limit(θ)_ in *Scn5a*^+/−^ RV epicardium and WT RV endocardium (from 16.57(17.89) to 28.95(23.61) ms; *U*= 37.0, *P*= 0.044, *r*= 0.48, and from 16.71(24.27) to 31.27(4.24) ms; *U*= 6.0, *P*= 0.027, *r*= 0.66). Flecainide therefore causes premature conduction failure in the *Scn5a*^+/−^ RV at higher pacing rates. Quinidine also results in early conduction failure in the RV of both *Scn5a^+/−^* and WT hearts.

### Conduction velocity alternans

In contrast with previous reports of smooth progressions of APD alternans with decreasing DI, θ′ alternans occurred abruptly before refractoriness. This precluded mono-exponential curve fitting and analytical comparisons of alternans to θ′ restitution curves. Magnitudes of θ′ alternans were thus compared at particular pacing rates (Fig. 4). At 8 Hz, corresponding to murine resting heart rates, there was no significant difference (χ^2^= 31.8, d.f. = 23, *P*= 0.104) in magnitude of θ′ alternans, which was close to zero in all conditions. However, at higher rates of 13.5 Hz, corresponding to exercising heart rates, flecainide treatment increased the magnitude of θ′ alternans specifically in the *Scn5a*^+/−^ RV epicardium (from 0.00(0.00) to 0.0051(2.55) ms^−1^, *P*= 0.021). This occurred shortly before the onset of refractoriness in this condition.

**Figure 4:**
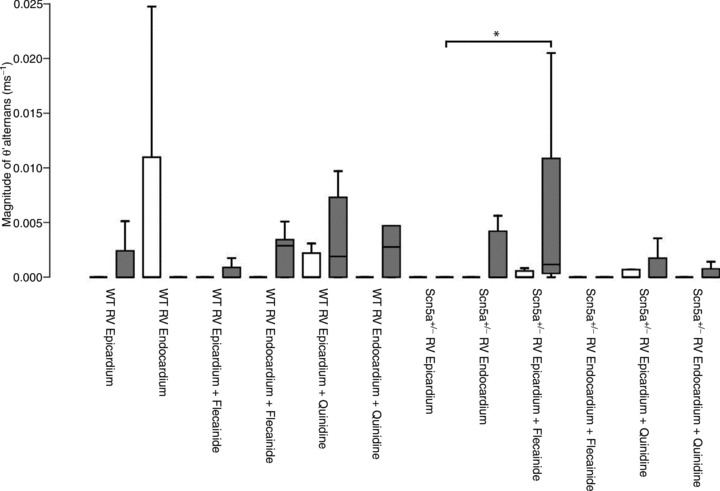
Box and whisker plots summarising the magnitude of θ ′ alternans in the RV at resting (8 Hz) (open plots) and exercising (13.5 Hz) (filled plots) heart rates Horizontal lines denote the Mdn, the box denotes the IQR, and the whisker the range. Little alternans occurred at resting heart rates with all Mdns being close to zero, and no significant differences between groups. However, the magnitude of θ′ alternans is markedly increased by high rate pacing. Significant (*P* < 0.05) increases in θ′ alternans was demonstrated in the *Scn5a*^+/−^ RV epicardium following flecainide challenge as denoted by the asterisk.

These findings demonstrate that θ′ alternans was marked in the flecainide-treated *Scn5a^+/−^* RV epicardium, which is the site previously implicated in the initiation of arrhythmia (Matthews *et al.* 2012), potentially suggesting their role in the arrhythmogenic mechanism. However, the contrast between the abruptness of the θ′ alternans function with the smooth θ′ restitution curve would preclude a direct causal relationship between them.

### Wavelength (λ) restitution

The latter findings prompted a unification of θ and APD parameters to give wavelength, which has previously been invoked as a major criterion for arrhythmia. θ and APD were combined to give λ′ and this is plotted against DI (Fig. 5). λ′ decreased non-linearly with DI, as reported previously (Weber, 2011; Lou *et al.* 2012). However, in common with θ′ restitution curves, further analysis was precluded by their differing dimensions and indirectly coupled variables (see Theory section).

**Figure 5:**
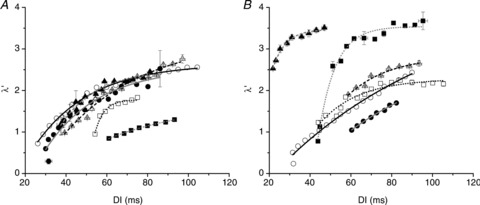
Plots of λ′ against DI Results obtained from typical (*N*= 1) WT (open symbols; black lines) and *Scn5a*^+/−^ (filled symbols; grey lines) hearts in the RV epicardium (*A*) and RV endocardium (*B*), before (circles; continuous line) and following addition of flecainide (squares; dashed line) or quinidine (triangles; dotted line). The data displayed represent hearts showing the Mdn value of λ′_max_ in the experimental group concerned.

Therefore λ′ was plotted against a similarly dimensioned λ′_0_, thereby permitting a feedback analysis (eqn (25), Theory section) in which instability takes place at a gradient of one. Figure 6 shows typical plots from WT and *Scn5a*^+/−^ RV, epicardium and endocardium, before and following treatment with flecainide and quinidine. The resulting curves progress together with considerably smaller discrepancy compared to Figs 3 or 5.

**Figure 6:**
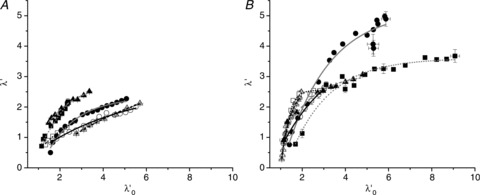
λ′ restitution curves plotting λ′ against λ′_0_ Results obtained from typical (*N*= 1) WT (open symbols; black lines) and *Scn5a*^+/−^ (filled symbols; grey lines) hearts in the RV epicardium (*A*) and RV endocardium (*B*), before (circles; continuous line) and following addition of flecainide (squares; dashed line) or quinidine (triangles; dotted line). The data displayed represent hearts showing the Mdn value of λ′_max_ in the experimental group concerned. Such curves fall closer together particularly at low λ′_0_ values than the plots shown in either Figs 3 or 5, or previously reported APD restitution curves. Values could differ at higher values of λ′_0_.

### Instability prediction using λ′ restitution

Importantly, there were no significant differences (χ^2^= 29.5, d.f. = 23, *P*= 0.166) in the predicted point of instability, λ′_0crit_, between WT or *Scn5a*^+/−^, RV or LV, or epicardial or endocardial values, whether before or following introduction of either flecainide or quinidine. This implies a convergence towards a common or minimally affected resting wavelength corresponding to the onset of positive feedback effects, potentially leading to arrhythmia.

The BCL at which the common value of λ′_0crit_ occurred, BCL_crit(λ)_, showed predictive differences in arrhythmic conditions. Flecainide increased BCL_crit(λ)_ specifically in *Scn5a*^+/−^ RV epicardium (from 59.86(30.94) to 95.67(12.37) ms; *U*= 11.0, *P*= 0.002, *r*= 0.66) (Fig. 7*A*). Quinidine increased BCL_crit(λ)_ in both WT RV endocardium and the *Scn5a*^+/−^ RV epicardium (from 33.54(56.48) to 100.18(52.88) ms; *U*= 3.0, *P*= 0.027, *r*= 0.72; and from 59.86(30.94) to 92.45(37.01) ms; *U*= 34.0, *P*= 0.033, *r*= 0.49, respectively).

**Figure 7:**
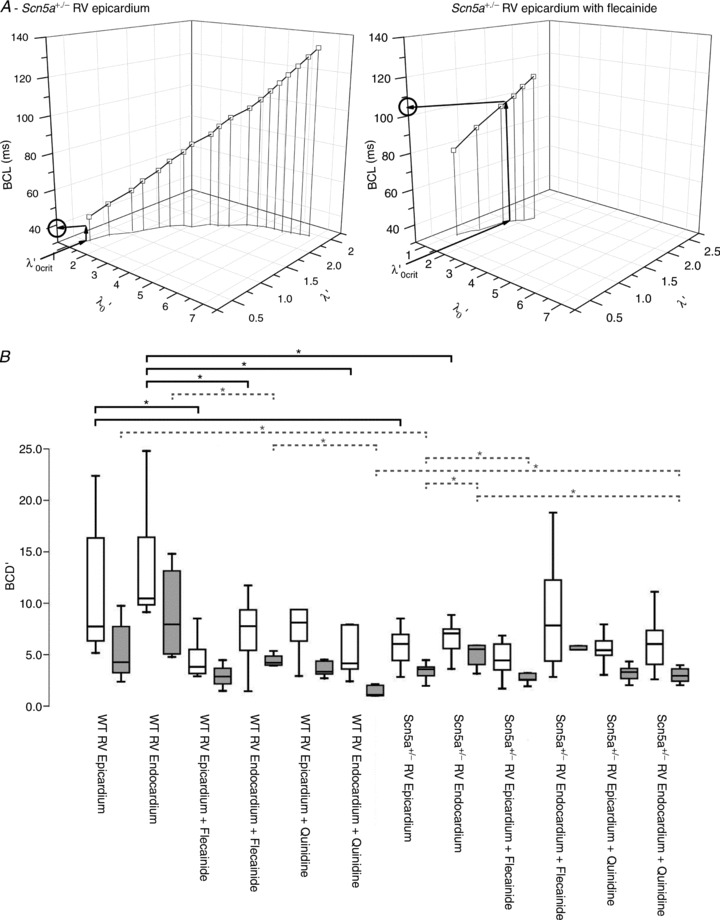
Instability prediction using λ′ restitution *A*, 3-D plots of λ′ restitution curves against the BCL in the *Scn5a*^+/−^ RV epicardium and *Scn5a*^+/−^ RV epicardium treated with flecainide, showing the BCL_crit(λ)_ (circled) corresponding to the λ′_0crit_ value (arrowed). BCL_crit(λ)_ was markedly increased by flecainide in the *Scn5a*^+/−^ RV epicardium despite similar λ′_0crit_ values. This was due to changes in the BCD′ shown in box and whiskers plots (*B*) at 8 Hz (open plots) and 13.5 Hz (filled plots) pacing in the RV. Horizontal lines indicate the Mdn, boxes the IQR, and whiskers show the range. *Significant differences, *P* < 0.05; continuous lines show comparisons between 8 Hz pacing, and dashed lines show comparisons between 13.5 Hz pacing.

The location on the λ restitution curve at any given pacing rate is given by the *total wavelength* (λ′+λ′_0_) or basic cycle distance BCD (BCD′= BCL/latency: Theory section). This may explain why a common instability point exists in spatial but not temporal terms. At a resting heart rate of 8 Hz (Fig. 7*B*), BCD′ in untreated *Scn5a*^+/−^ RV epicardium and endocardium were smaller than in the corresponding WT (6.03(2.66) *vs*. 7.74(10.1); *U*= 144.5, *P*= 0.001, *r*= 0.50 and 7.06(2.58) *vs*. 13.8(12.67); *U*= 8.0, *P*= 0.009, *r*= 0.66, respectively). Quinidine decreased BCD′ in the WT RV endocardium (from 13.8(12.67) to 6.24(4.48); *U*= 5.0, *P*= 0.014, *r*= 0.71). At an exercising heart rate of 13.5 Hz (Fig. 7*B*), BCD′ in untreated *Scn5a^+/−^* RV epicardium was smaller than in WT RV epicardium (3.42(0.77) *vs*. 5.20(4.62); *U*= 117.0, *P*= 0.024, *r*= 0.42). There was also an epicardial–endocardial difference in the *Scn5a*^+/−^ RV (3.42(0.77) *vs*. 5.27(1.88); *U*= 23.0, *P*= 0.001, *r*= 0.67). Flecainide reduced the BCD′ in the *Scn5a*^+/−^ RV epicardium (from 3.42(0.77) to 2.66(0.82); *U*= 20.0, *P*= 0.030, *r*= 0.48). Quinidine exerted marked actions especially in the WT. In particular, the BCD′ in the quinidine-treated WT RV endocardium was smaller than in the corresponding *Scn5a^+/−^* (1.09(1.06) *vs*. 3.00(1.94); *U*= 2.0, *P*= 0.047, *r*= 0.73). Finally, the BCD′ at the λ′_0crit_ showed no significant differences (χ^2^= 29.7, d.f. = 23, *P*= 0.158) between genotype, region or pharmacological condition, giving a consistent endpoint at a BCD′ of 2.41(3.62).

### The limiting features of λ′ restitution curves

Maximum values of excitation wavelength at low heart rates, λ′_max_, showed endocardial–epicardial differences in the untreated WT LV (8.16(12.35) *vs*. (3.08(2.25); *U*= 32.0, *P*= 0.041, *r*= 0.49) and untreated WT RV (4.11(1.38) *vs*. 2.78(1.02); *U*= 16.0, *P*= 0.048, *r*= 0.49). In the untreated *Scn5a*^+/−^, endocardial–epicardial λ′_max_ differences were present only in the RV (5.25(2.18) *vs*. 2.32(0.92); *U*= 37.0, *P*= 0.021, *r*= 0.49). λ′_max_ was greater in the WT LV endocardium than in the corresponding *Scn5a*^+/−^ (8.16(12.35) *vs*. (2.10(0.96); *U*= 6.0, *P*= 0.007, *r*= 0.72). Quinidine decreased λ′_max_ in the WT RV endocardium (from 4.11(1.38) to 2.61(0.41); *U*= 2.0, *P*= 0.018, *r*= 0.76) and WT LV endocardium (from 8.16(12.35) to 2.00(2.08); *U*= 9.0, *P*= 0.048, *r*= 0.59). No λ′_max_ differences were observed in flecainide-treated conditions. In untreated hearts λ′_max_ was thus greater in the endocardium than the epicardium. This is probably present to prevent back conduction from the epicardium, but results in heterogeneity existing across the myocardial wall. Flecainide and quinidine both reduce this effect by reducing endocardial λ′_max_.

At high pacing rates, the maximum gradient, *m*_max(λ)_, of the λ′ restitution curve typically took a value of 1.7(3.45), exceeding a value of one. There were few differences in the value of λ′_0_ at which λ′ was zero, λ′_0limit_. This was greater only in the flecainide-treated WT RV endocardium compared to the corresponding epicardium (1.13(0.34) *vs*. 0.67(0.40); *U*= 10.0, *P*= 0.041, *r*= 0.44). Conversely the BCL at the λ′_0limit_ was not significantly different in any group (χ^2^= 31.4, d.f. = 23, *P*= 0.114). Thus both *m*_max(λ)_, or λ′_0limit_ largely assumed similar values under arrhythmic and non-arrhythmic conditions consistent with high rate convergence of λ restitution curves.

### The dependence of λ alternans on λ′_0_

Plots of the magnitude of λ′ alternans against λ′_0_ (Fig. 8) were concordant with the λ restitution plots. In parallel with λ′_0crit_ in the λ restitution curves, there were no observed differences (χ^2^= 23.7, d.f. = 23, *P*= 0.420) between the values of λ′_0_ that corresponded to a gradient of –1 (λ′_0crit(alt)_) between conditions. Furthermore, there were no significant differences (χ^2^= 29.0, d.f. = 23, *P*= 0.180) in maximum gradient (*m*_maxλ(alt)_). However, differences could exist in the BCL at which the λ′_0crit(alt)_ occurred (BCL_critλ(alt)_). These differences took place in the *Scn5a^+/−^* RV epicardium following addition of either flecainide (from 57.80(42.27) to 97.87(8.19) ms; *U*= 0.0, *P*= 0.003, *r*= 0.68) or quinidine (from 57.80(42.27) to 96.40(3.17) ms; *U*= 5.0, *P*= 0.009, *r*= 0.61), in accord with the λ restitution changes noted in BCL_crit(λ)_. Additionally BCL_critλ(alt)_ was greater in the *Scn5a^+/−^* LV epicardium as compared to the corresponding LV endocardium (57.86(15.34) *vs*. 7.54(10.47); *U*= 1.0, *P*= 0.024, *r*= 0.71).

**Figure 8:**
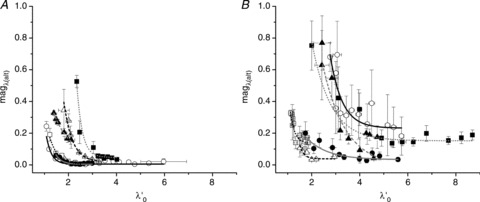
The dependence of λ alternans on λ Typical plots of the magnitude of λ′ alternans, mag_λ(alt)_ against λ′_0_ in WT (open symbols; black lines) and *Scn5a*^+/−^ (filled symbols; grey lines), RV epicardium (*A*), and RV endocardium (*B*), before (circles; continuous line) and following addition of either flecainide (squares; dashed lines) or quinidine (triangles; dotted lines). Data represent the Mdn value of λ′_0crit(alt)_ as all curves progress together with common negative gradients.

The values of λ′_0_ at which the λ′ alternans plots and λ restitution plots intersect (λ′_0χ_) corresponds to alternate loss of propagating waves. λ′_0χ_ was higher in the presence of flecainide in the WT RV endocardium (1.41(0.68)) than in the corresponding epicardium (0.87(0.32); *U*= 2.0, *P*= 0.042, *r*= 0.74). λ′_0χ_ was decreased by quinidine in the WT LV epicardium (from 1.33(0.06) to 0.99(0.37); *U*= 14.0, *P*= 0.024, *r*= 0.60). This suggests differences in the onset of block caused by alternans following addition of Na^+^ channel blocker. Despite the *Scn5a*^+/−^ RV epicardium showing similar values of λ′_0χ_, before and following addition of flecainide, this took place at a significantly greater BCL (from 53.11(22.25) to 83.28(19.97), *U*= 17.0, *P*= 0.029, *r*= 0.52), consistent with an earlier occurrence of conduction block resulting from λ alternans.

### Concurrence between λ alternans and λ restitution

Plots of the slopes of the λ′ restitution functions (*m*_λ_) against the magnitude of λ′ alternans (mag_λ(alt)_) were analytically assessed using an equation of the form mag_λ(alt)_=*qm*_λ_^s^+*C*, where *q*, *s* and *C* are defined in the Methods. Values of *q* (χ^2^= 29.7, d.f. = 23, *P*= 0.159) and *s* (χ^2^= 24.6, d.f. = 23, *P*= 0.372) showed no significant differences between conditions. *C*, or the background level of alternans (*y*_0λ(alt)_), was marginally increased in the WT RV epicardium following quinidine treatment (from 0.01(0.01) to 0.03(0.02); *U*= 20.0, *P*= 0.048, *r*= 0.048). This gave median values for *q*, *s* and *C* of 0.33, 2.6 and 0.013, respectively, resulting in the function:


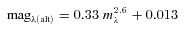


This function was directly compared with that obtained for APD restitution (Matthews *et al.* 2012) which had suggested that the magnitude of APD alternans was given by:


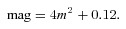


Both these functions demonstrated that the magnitude of alternans non-linearly increased with restitution gradient. However, Fig. 9 shows that when plotted over a physiological range between restitution slopes of 0 to *m*_max_, the λ′ restitution relationship showed a closer adherance and converged towards the line of equality. In contrast, APD restitution significantly underestimated the magnitude of alternans and diverged from equality, suggesting that a function which includes θ is superior to one employing APD alone. Nevertheless, λ′ restitution predicted a slightly greater alternans than was observed and *m*_max(λ)_ was able to just exceed a value of one, where chaotic feedback should occur. This implicates one or more factors additional to θ and APD which may damp the observed alternans at high rates.

**Figure 9:**
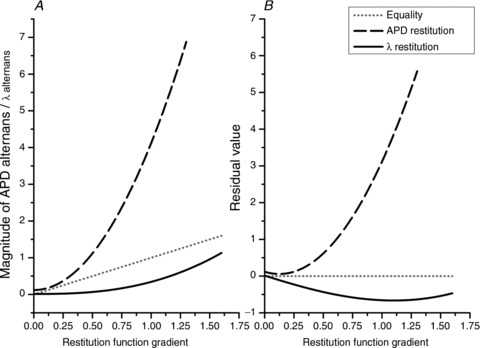
Comparison of the relationships between the APD restitution gradient and the magnitude of APD alternans taken from previous reports (Matthews *et al.* 2012) and between the λ ′ restitution gradient and the magnitude of λ ′ alternans This demonstrates that between a gradient of 0 and *m*_max_ for either function the λ′ restitution offers a better concordance with (*A*), and a smaller discrepancy from (*B*), the equality line (*y*=*x*). In contrast, the APD alternans rapidly diverges away from (*A*), and gives a larger discrepancy from (*B*), the equality line, excluding a direct relationship.

## Discussion

The Brugada syndrome is an autosomal dominant condition most commonly associated with mutations in the *SCN5A* gene, encoding the voltage-gated Na^+^ channel Na_v_1.5 (Alings & Wilde, 1999). This mediates the AP upstroke and its myocardial propagation. BrS is associated with a propensity to fatal ventricular arrhythmias, particularly polymorphic VT and VF (Brugada & Brugada, 1992). BrS is clinically characterised by a type I ECG pattern involving a right bundle branch block, as well as ST segment elevation and T wave inversion in the right precordial leads (Wilde *et al.* 2002). Other type II and type III patterns, although also associated with BrS, are not pathognomic but may be converted to the type I pattern through diagnostic flecainide challenge (Shahrzad *et al.* 2011). In contrast to previous guidelines, recent reports have suggested that invasive electrophysiological testing involving induction of arrhythmia using imposed pacing does not provide a consistent risk assessment (Priori *et al.* 2002; Paul *et al.* 2007), demonstrating an incomplete knowledge of the pathophysiology. Implantable cardioverter defibrillator (ICD) installation remains the definitive management following ECG diagnosis with/without family history, although quinidine may offer an adjunctive therapy (Belhassen *et al.* 2004).

A better understanding of the mechanisms underlying arrhythmogenicity in BrS would therefore aid diagnosis and treatment. The use of *Scn5a* haploinsufficient murine models enables controlled studies to this end. *Scn5a*^+/−^ hearts show a 50% reduction in RV Na_v_1.5 expression (Martin *et al.* 2012*b*; Fig. 10*A*), and replicate many BrS features. This includes a greater arrhythmic tendency particularly following flecainide challenge (Stokoe *et al.* 2007; Matthews *et al.* 2012).

**Figure 10:**
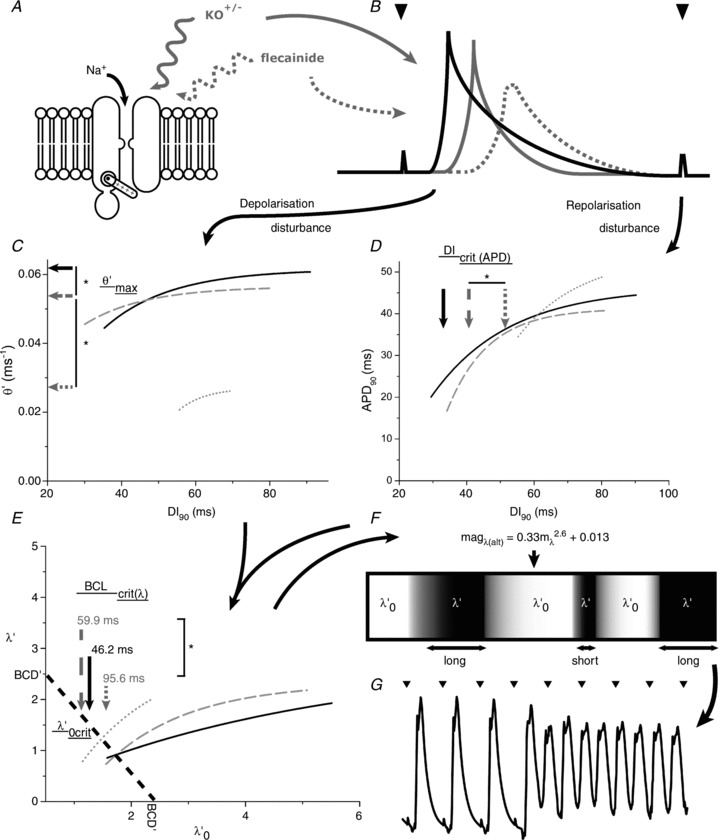
Summary of the concept of wavelength restitution analysis exemplified by the murine *Scn5a*^+/−^ system following flecainide *A*, illustration of the voltage-gated Na^+^ channel, alterations in which ultimately form the basis of the arrhythmic tendency in hearts showing a 50% Na^+^ channel reduction (KO) in the RV. *B*, comparison of the consequent AP waveforms in untreated WT (continuous black lines), untreated *Scn5a*^+/−^ (dashed grey lines) and flecainide-treated *Scn5a*^+/−^ (dotted grey lines). The *Scn5a*^+/−^ RV epicardium shows longer latencies and shorter APs when compared to WT. Flecainide substantially increases latency but also increases APD. These differences demonstrate depolarisation and repolarisation disturbances that are rate dependent and assessed by conduction velocity (*C*) and APD restitution curves (*D*). *C*, right-sided conduction block exists in untreated *Scn5a^+/−^* hearts compared to WT. This is exacerbated by flecainide. *D*, data taken from Matthews *et al.* (2012) demonstrates that untreated *Scn5a^+/−^* and WT hearts have similar DI_crit(APD)_ values. However, flecainide markedly increases this value in *Scn5a^+/−^* RV epicardium. *E*, both depolarisation and repolarisation disturbances unify to the λ restitution curve. These may show differences in maximum value but converge to a common point of instability λ′_0crit_. However, the pacing rate required to achieve this point is significantly lower in the *Scn5a^+/−^* RV epicardium when treated with flecainide. *F*, the gradients of the λ′ restitution curve map to the magnitude of λ′ alternans through a simple analytical equation which shows greater linearity over physiological heart rates than previously described for APD restitution. This results in alternating excitation wavelengths that may give rise to heterogeneities or risk of re-entry around an existing heterogeneity, and consequent re-entrant arrhythmia in the form of VT (*G*).

Previous reports had quantified the onset of APD and AP amplitude alternans with increasing heart rates. Alternans became more prominent with increasing pacing rate, becoming sustained in the presence of flecainide in the *Scn5a*^+/−^ RV epicardium at lower rates than in other recording sites or in the WT (Matthews *et al.* 2010). More extensive comparisons of arrhythmic properties demonstrated that untreated *Scn5a*^+/−^ but not WT hearts showed significant incidences of VT at low pacing rates. When challenged with flecainide, *Scn5a*^+/−^ hearts showed greater incidences of VF than WT at higher heart rates. In *Scn5a*^+/−^ hearts, quinidine decreased the incidence of VT at low rates and slightly increased the incidence of VF at high rates. However, quinidine was markedly pro-arrhythmic in the WT at all heart rates. In parallel with the arrhythmogenicity observed at high rates, APD restitution curves from flecainide-challenged *Scn5a*^+/−^ RV epicardium showed increased DI_crit_ (Fig. 10*D*). This was accompanied by increased magnitudes of APD alternans. However, the relationship between APD alternans and restitution slope through different pacing rates was non-linear and consistently underestimated the extent of alternans (Matthews *et al.* 2012). This discrepancy potentially explains the varied success of restitution analysis in the prediction of arrhythmic tendency in clinical situations (Narayan *et al.* 2007*a*,*b*; Dorenkamp *et al.* 2013). They indicated that whilst relationships between APD restitution and alternans may exist, other factors that could include θ might also contribute.

The present study accordingly examined the effect of varying heart rates on θ under conditions otherwise identical to those explored in the previous reports. It then introduced a novel, more comprehensive, wavelength restitution analysis of the likelihood of AP wavebreak (summarised in Fig. 10).

Alterations in conduction latencies reflecting θ were characterised at decreasing BCLs and consequently DIs (Fig. 10*B*). Classical indicators of the broadness of the θ restitution function (Bursac & Tung, 2006; Qu *et al.* 2006) constituted poor indicators of the reported arrhythmia and alternans. However, the maximum achievable θ′ and the DI at which conduction failed correlated well with arrhythmic tendency, highlighting the *Scn5a*^+/−^ RV epicardium both before and following flecainide challenge (Fig. 10*C*). θ′ alternans was much less frequent than previously reported for APD alternans, showing an abrupt onset at pacing rates presaging refractoriness. Flecainide treatment increased θ′ alternans in *Scn5a*^+/−^ RV epicardium, in accord with the changes in maximum θ′ and conduction failure.

APD and θ′ were then combined to yield a non-linear reduction of *excitation* wavelength with falling DI, similar to those constructed in previous reports (Weber, 2011; Lou *et al.* 2012). However, use of DI, dimensioned in time, did not permit a feedback analysis against a distance, λ. Prediction of instabilities using restitution analysis requires variables that both show reciprocal effects upon each other and are identically dimensioned. This was achieved by a further translation of the DI into a *resting* wavelength, given by the product of θ′ and DI, yielding a novel λ restitution function (Fig. 10*E*).

At low heart rates, these could show differing, epicardial and endocardial, maximum values of λ within the untreated hearts. Quinidine decreased the WT endocardial maximum λ and abolished the *Scn5a*^+/−^ endocardial–epicardial difference, consistent with its pro-arrhythmic effects in WT but anti-arrhythmic effects in *Scn5a*^+/−^ (Matthews *et al.* 2012). The novel λ restitution theory, like classical APD restitution, predicted that with increasing heart rate, unstable positive feedback would lead to alternans, failure of wave propagation and re-entrant foci. Positive feedback would occur at a λ restitution gradient of one. Strikingly, this took place at indistinguishable resting (λ′_0crit_) and total (BCD) wavelengths, in both genotypes, all recording sites and whether before or following addition of either flecainide or quinidine. This finding suggests a common point for the onset of instability. The pacing rates at which this point was achieved were indistinguishable in untreated hearts; however, they were decreased by flecainide and quinidine specifically in the *Scn5a*^+/−^ RV epicardium, and by quinidine in the WT RV endocardium. Such a difference arises because the BCD′ at any given pacing rate was reduced both by the *Scn5a*^+/−^ genotype and by the further addition of flecainide in the RV epicardium (Fig. 10*E*). BCD′ was also reduced by quinidine in the WT RV endocardium. As BCD′ is given by the product of the BCL and θ′, these decrements are attributable to reductions in θ as BCL was experimentally controlled. However, heart rate would be an important factor *in vivo*.

The corresponding λ alternans also showed common critical resting wavelengths regardless of genotype, recording sites or conditions. Furthermore, this critical point occurred at higher pacing rates in the *Scn5a*^+/−^ RV epicardium following addition of either flecainide or quinidine. Both these findings are in direct agreement with the λ restitution results. Over the range of heart rates that were studied, the gradient of the λ restitution function agreed with the magnitude of its alternans more strongly than did APD restitution (Fig. 10*F*). Thus, accounting for changes in conduction velocity by using λ restitution is probably closer to the mechanistic basis of alternans, and is therefore a superior predictor of arrhythmia (Fig. 10*G*). Common instability points on λ restitution curves implicate particular intrinsic cellular mechanisms controlling the relationship between excitation and resting wavelengths. This could reflect the existence of either interrelationships between channel opening and voltage changes (Watanabe *et al.* 2001), or other signalling mechanisms which include channel modulation, potentially through alterations in Ca^2+^ (Tohse, 1990) and ATP homeostasis, resulting in a fixed product of APD and upstroke. Whilst there was much less discrepancy between the λ restitution function and alternans, λ restitution provided a small overestimate of the alternans observed and the maximum gradient of the function was able to exceed one, in contrast to the underestimate obtained by APD restitution. This probably reflects the superiority of accounting for changes in θ but suggests the existence of a small mitigating factor. Other processes may therefore damp the magnitude of alternans. One such candidate may be intracellular Ca^2+^ at normal or increased levels, as this is known to modulate *I*_Na_ (Casini *et al.* 2009), *I*_Ca_ (Isaev *et al.* 2004) and gap junctions (Lazrak *et al.* 1994).

The present findings were obtained in intact murine hearts (Sabir *et al.* 2008) permitting direct study of genetic channelopathy and their response to drugs, without prior pharmacological induction (Yan & Antzelevitch, 1999). Murine hearts show clinically relevant arrhythmias (Vaidya *et al.* 1999). In common with human hearts, their ventricular excitation depends on electrotonic spread of a membrane depolarisation, driven by *I*_Na_ (Na_v_1.5) and *I*_Ca(L)_ (Ca_v_1.2) (Guo *et al.* 1999), which is repolarised by outward K^+^ currents (Noble, 1979). Murine fast and slow inactivating transient outward K^+^ currents, *I*_to,f_ and *I*_to,s_ have more prominent roles, resulting in shorter AP durations. Furthermore, the function of the rapid delayed rectifier K^+^ current, *I*_Kr_, and the slow delayed rectifier K^+^ current, *I*_Ks_, in late repolarisation is replaced by a greater noninactivating steady-state voltage activated K^+^ current *I*_ss_ (Babij *et al.* 1998; Drici *et al.* 1998; Balasubramaniam *et al.* 2003) and slowly inactivating K^+^ current, *I*_Kslow_, contributions (Boyle & Nerbonne, 1992; Boyett *et al.* 1998; Danik *et al.* 2002; Sabir *et al.* 2008). Mouse and human hearts show similar conduction architecture (Miquerol *et al.* 2004; Gaborit *et al.* 2007; Remme *et al.* 2009) with the exception of one discontinuity in the connective tissue isolating the common bundle from septal working myocardium (van Rijen *et al.* 2001). However, the present studies found no evidence for major conduction discontinuities with changes in heart rate, which would result in alterations in the θ restitution curves (Derksen *et al.* 2003). These differences remain compatible with AP mechanisms that similarly involve excitation and recovery wavelengths permitting wavebreak phenomena, although absolute values may differ between mouse and human hearts.

The experiments were performed in Langendorff-perfused preparations which provide well established (Skrzypiec-Spring *et al.* 2007; Bell *et al.* 2011) platforms for the investigation of intrinsic cardiac properties that could be paced incrementally. MAP recordings offered a stable (Yang & Kittnar, 2010) means of locally recording waveform latency and repolarisation with high fidelity (Franz, 1999; Knollmann *et al.* 2001). Theoretical modelling studies confirm that MAP recovery time courses closely correlate with transmembrane AP recordings in an anisotropic bidomain myocardial surface (Franzone *et al.* 2007). MAP recordings do not reflect absolute amplitudes or upstroke gradients but these values were not required in the current analysis. MAP electrodes were mounted on a spring-based support that kept the inactivating pole of the MAP electrode firmly in contact with the myocardium. Additionally the compressibility of the MAP sponge prevented loss of contact with the local extracellular fluid. Both of these factors maintained the interelectrode distance and thus viability of the MAP recordings despite cardiac contractions. MAPs have been extensively used in recent clinical and experimental studies (Nishii *et al.* 2010; Ashino *et al.* 2011; Choi *et al.* 2011). The experiments tested the effects of two pharmacological agents in current diagnostic or therapeutic use for BrS. The major and desired effect of flecainide is a slow block of the Na_v_1.5 channel. It also blocks *I*_Kr_ but this current is not present in murine hearts (Wang *et al.* 1996). Recent studies suggest actions blocking the open state of the ryanodine receptor RyR2 resulting in reduced peak Ca^2+^ release. However, unlike other RyR2 blockers such as tetracaine, flecainide does not affect the transition from resting to open state, and thus does not affect RyR2 opening frequency or Ca^2+^ store levels (Hilliard *et al.* 2010). At high concentrations in excess of those studied here, flecainide blocks all K^+^ channels (Slawsky & Castle, 1994). Quinidine also blocks Na_v_1.5 with intermediate kinetics, but shows major off-target effects in blocking *I*_to_ (Yatani *et al.* 1993), in addition to *I*_Kr_ and *I*_Ks_ (Yang & Roden, 1996). The present differential effects of these drugs are therefore explained by the additional *I*_K_ blocking activity of quinidine. Both drugs slow conduction velocity due to reducing *I*_Na_. This reduces the basic cycle distance. It does so markedly in the *Scn5a^+/−^* RV epicardium, which is most susceptible to these agents due to its particularly reduced Na_v_1.5 channel density (Martin *et al.* 2012*b*). Flecainide exerted less pronounced effects on the WT, due to a higher reserve of Na_v_1.5, particularly in the RV. However quinidine could exert pro-arrhythmic effects in the WT, particularly the RV endocardium. This is probably due to changes in endocardial wavelength at lower pacing rates caused by K^+^ channel blockade, in conjunction with reductions in conduction velocity. The *Scn5a^+/−^* is partially protected from this effect due to the greater relative importance of unopposed repolarising currents (Martin *et al.* 2012*a*).

The present findings have implications for arrhythmia management involving rate and rhythm control of cardiac activity. The CAST trial demonstrated that use of flecainide increased mortality in ischaemic heart disease patients despite the beneficial effects of reducing ectopic events, therefore leading to recommendations for selecting rate control in arrhythmia management (Echt *et al.* 1991). The present results suggest that agents which reduce θ should be avoided in tachycardic hearts or measures should be taken to avoid tachycardia when these drugs are administered, to prevent reductions in total wavelength (BCD). This could be achieved by either use of β-blockers or the newer pacemaker current, *I*_f_ blockers, of which the latter would avoid negative inotropic effects. Novel drug development directed at rhythm control should seek to do so without reducing θ.

## Translational perspective

Arrhythmias are a common cause of death, resulting from genetic conditions such as Brugada syndrome or acquired conditions such as ischaemic heart disease. Current diagnostic procedures have a low predictive value, often relying on patient and family history and random ECG testing. Invasive electrophysiological studies using artificial pacing to induce arrhythmia can be used in risk stratification but have recently been withdrawn from the Brugada syndrome management guidelines due to lack of predictive value. Furthermore, characterisation of arrhythmic parameters such as APD restitution have also had mixed success. These stem from incomplete knowledge of the pathophysiology of arrhythmias, which currently considers repolarisation and depolarisation separately. The current study provides novel physiological concepts underlying the generation of alternans and arrhythmia. The resulting wavelength restitution and alternans properties were perturbed in mouse hearts modelling Brugada syndrome and could be used as biomarkers in human invasive or ECG studies. However, their use is likely to be applicable to all arrhythmic conditions. More accurate biomarkers could also be used in drug development, aiding both discovery of new anti-arrhythmic agents and excluding potentially dangerous ones more effectively than current HERG and QT prolongation assays. Additionally, the present results suggest that drug screening for effects on conduction velocity via blockade of the Na_v_1.5 channel would be advisable in mainstream drug safety profiling. Finally, they suggest that agents that reduce heart rate or increase conduction velocity might be useful in anti-arrhythmic therapy, probably reflecting the empirical superiority of current drugs controlling rate in preference to rhythm.

## Abbreviations

AP: action potential
APD: action potential duration
BCD: basic cycle distance
BCL: basic cycle length
BrS: Brugada syndrome
DI: diastolic interval
ERP: effective refractory period
IQR: interquartile range
LV: left ventricle
MAP: monophasic action potential
Mdn: median
RP: refractory period
RV: right ventricle
VF: ventricular fibrillation
VT: ventricular tachycardia
WT: wild-type

## Supplementary material

Supplementary Figures 1–6

Supplemental data section 6

Supplemental data section 7
