# Supplementary material for: Action potential wavelength restitution predicts alternans and arrhythmia in murine Scn5a+/− hearts
Source: J Physiol. 2013 Jul 8;591(Pt 17):4167–88. doi: 10.1113/jphysiol.2013.254938 (PMC3779110; doi:10.1113/jphysiol.2013.254938)
Supplement: Supplementary file 1 [file tjp0591-4167-SD1.pdf]

TABLES

Table 1 **-** Results of θ' restitution analysis. Data displayed as medians (Mdn) and interquartile ranges (IQRs) for all groups studied. N is the number of hearts.

		***Latency at ERP* (ms)**		**τ_θ (ms)**		**$DI_{limit(\theta)}$ (ms)**		**θ'_{max} (ms^{-1})**		**$m_{max(\theta)}$ (ms^{-2})**	
	**N**	**Mdn**	**IQR**	**Mdn**	**IQR**	**Mdn**	**IQR**	**Mdn**	**IQR**	**Mdn**	**IQR**
WT LV epicardium	17	18.43	13.35	16.12	29.18	12.70	21.10	0.102	0.064	0.0011	0.0027
WT RV epicardium	18	18.31	6.99	17.77	8.86	12.40	15.06	0.066	0.026	0.0010	0.0015
WT LV endocardium	8	21.06	11.77	6.91	5.22	24.80	5.32	0.058	0.022	0.0030	0.0020
WT RV endocardium	11	19.01	16.46	33.60	44.56	16.71	24.27	0.083	0.017	0.0010	0.0041
WT LV epicardium + flecainide	6	40.31	13.34	72.67	50.90	24.90	22.78	0.083	0.016	0.0010	0.0021
WT RV epicardium + flecainide	6	49.34	9.79	17.86	25.90	49.70	20.76	0.038	0.025	0.0007	0.0003
WT LV endocardium + flecainide	9	19.33	13.17	27.77	26.43	16.84	23.21	0.093	0.042	0.0013	0.0005
WT RV endocardium + flecainide	10	21.72	10.13	16.60	23.59	31.43	20.85	0.076	0.039	0.0022	0.0074
WT LV epicardium + quinidine	7	39.94	12.23	23.82	33.69	27.71	32.14	0.044	0.036	0.0007	0.0009
WT RV epicardium + quinidine	7	25.89	8.88	25.60	24.95	25.70	14.95	0.084	0.030	0.0023	0.0044
WT LV endocardium + quinidine	6	36.91	12.93	40.10	34.81	23.40	11.64	0.070	0.034	0.0008	0.0007
WT RV endocardium + quinidine	6	41.41	12.89	13.08	8.89	31.27	4.24	0.036	0.023	0.0012	0.0008
Scn5a$^{+/-}$ LV epicardium	13	27.15	8.01	14.74	4.99	15.66	11.43	0.064	0.008	0.0009	0.0006
Scn5a$^{+/-}$ RV epicardium	18	24.59	6.68	16.98	14.41	16.57	17.89	0.055	0.011	0.0008	0.0008
Scn5a$^{+/-}$ LV endocardium	7	23.86	8.36	20.00	29.40	9.52	46.66	0.062	0.018	0.0006	0.0007
Scn5a$^{+/-}$ RV endocardium	8	16.74	9.20	17.86	20.70	14.55	11.75	0.076	0.032	0.0014	0.0022
Scn5a$^{+/-}$ LV epicardium + flecainide	7	29.40	15.10	8.13	4.84	39.77	22.54	0.050	0.009	0.0018	0.0032
Scn5a$^{+/-}$ RV epicardium + flecainide	7	38.20	18.94	9.53	3.39	44.66	22.16	0.027	0.028	0.0047	0.0047
Scn5a$^{+/-}$ LV endocardium + flecainide	5	35.69	28.30	8.20	3.72	29.60	23.73	0.081	0.058	0.0032	0.0134
Scn5a$^{+/-}$ RV endocardium + flecainide	7	25.81	19.75	11.42	10.23	40.10	18.99	0.069	0.060	0.0033	0.0205
Scn5a$^{+/-}$ LV epicardium + quinidine	8	30.49	14.28	16.97	26.57	21.71	24.46	0.048	0.020	0.0008	0.0005
Scn5a$^{+/-}$ RV epicardium + quinidine	10	31.88	12.02	13.89	11.35	28.95	23.61	0.052	0.021	0.0014	0.0009
Scn5a$^{+/-}$ LV endocardium + quinidine	5	29.40	11.04	10.55	6.76	33.57	8.74	0.049	0.010	0.0012	0.0006
Scn5a$^{+/-}$ RV endocardium + quinidine	9	26.19	21.21	12.15	11.66	21.52	30.31	0.057	0.012	0.0006	0.0005

Table 2 - Results of λ' restitution analysis. Data displayed as medians (Mdn) and interquartile ranges (IQRs) for all groups studied. N is the number of hearts.

		τ_λ		λ'_{0crit}		λ'_{0limit}		λ'_{max}		$m_{max(\lambda)}$		$BCL_{crit(\lambda)}$ (ms)		$BCD'_{crit(\lambda)}$	
	**N**	**Mdn**	**IQR**	**Mdn**	**IQR**	**Mdn**	**Mdn**	**IQR**	**IQR**	**Mdn**	**IQR**	**Mdn**	**IQR**	**Mdn**	**IQR**
WT LV epicardium	14	1.98	2.05	1.46	0.81	0.71	0.26	3.08	2.25	1.85	0.95	68.49	25.00	3.00	1.96
WT RV epicardium	15	2.05	1.71	1.15	1.31	0.60	0.43	2.78	1.02	1.02	1.23	46.23	38.92	2.10	2.55
WT LV endocardium	11	2.84	10.24	2.00	7.95	0.88	0.79	8.16	12.35	3.19	4.92	83.02	41.06	4.12	15.12
WT RV endocardium	6	4.07	3.26	1.05	3.50	0.63	0.37	4.11	1.38	1.06	0.85	33.54	56.48	2.40	6.39
WT LV epicardium + flecainide	7	1.32	2.45	0.94	1.96	0.66	0.70	1.97	2.56	1.56	1.97	74.10	60.88	1.89	3.54
WT RV epicardium + flecainide	6	1.73	1.01	1.03	0.88	0.67	0.40	2.04	0.57	1.58	5.37	77.40	34.24	1.55	1.88
WT LV endocardium + flecainide	10	3.81	5.72	1.11	8.15	1.71	1.83	5.06	3.21	1.13	2.50	66.65	81.03	1.46	13.31
WT RV endocardium + flecainide	7	2.28	1.99	1.47	1.16	1.13	0.34	2.52	0.66	1.71	5.32	67.72	35.63	2.32	4.01
WT LV epicardium + quinidine	9	2.22	2.94	0.73	1.86	0.48	0.71	2.42	1.16	1.04	0.67	69.14	52.88	1.26	4.30
WT RV epicardium + quinidine	10	2.74	2.22	1.14	0.72	0.73	0.13	3.18	1.47	1.03	0.51	53.77	27.05	1.49	1.39
WT LV endocardium + quinidine	6	2.43	3.24	0.83	2.81	0.77	0.84	2.00	2.08	0.97	1.34	91.24	17.35	1.19	4.91
WT RV endocardium + quinidine	8	1.26	1.39	1.80	0.35	0.99	0.11	2.61	0.41	9.00	42.48	100.18	52.36	3.00	1.63
$Scn5a^{+/-}$ LV epicardium	13	1.99	0.80	1.90	1.13	0.71	0.23	3.47	1.99	2.18	1.12	63.75	19.57	3.03	2.50
$Scn5a^{+/-}$ RV epicardium	23	1.54	1.48	1.09	1.14	0.73	0.65	2.32	0.92	1.30	4.96	59.86	30.94	1.90	2.03
$Scn5a^{+/-}$ LV endocardium	8	1.90	1.86	1.11	2.68	1.17	1.27	2.10	0.96	1.77	53.16	62.43	54.57	1.88	4.39
$Scn5a^{+/-}$ RV endocardium	9	1.33	1.52	1.71	1.38	1.09	0.32	5.25	2.18	3.67	4.74	65.69	40.38	4.04	10.46
$Scn5a^{+/-}$ LV epicardium + flecainide	6	1.27	0.74	2.02	1.17	1.07	0.92	2.86	0.58	2.08	3.77	92.80	28.01	3.00	2.60
$Scn5a^{+/-}$ RV epicardium + flecainide	9	0.81	0.79	1.72	0.65	0.95	0.17	2.11	0.92	3.47	7.65	95.67	12.37	2.76	1.90
$Scn5a^{+/-}$ LV endocardium + flecainide	8	1.51	1.44	2.00	1.56	1.38	1.39	2.22	2.24	3.02	12.49	104.57	115.89	3.62	3.60
$Scn5a^{+/-}$ RV endocardium + flecainide	6	3.74	7.14	0.26	7.60	0.51	1.45	4.58	5.27	1.70	5.37	70.79	28.44	2.05	9.86
$Scn5a^{+/-}$ LV epicardium + quinidine	7	2.09	0.74	1.37	1.07	0.73	0.52	2.84	0.73	1.80	2.47	61.64	54.39	2.56	2.17
$Scn5a^{+/-}$ RV epicardium + quinidine	9	1.06	2.06	1.52	0.53	0.80	0.34	2.65	0.77	2.68	9.79	92.45	37.01	3.00	1.48
$Scn5a^{+/-}$ LV endocardium + quinidine	6	1.11	1.90	2.65	2.00	1.50	1.21	2.49	0.82	18.30	81.13	118.16	37.45	3.84	3.17
$Scn5a^{+/-}$ RV endocardium + quinidine	9	2.02	1.07	1.51	1.06	0.79	0.94	3.36	2.78	1.51	1.56	60.32	33.69	2.74	2.99

Table 3 - Results of λ' alternans analysis. Data displayed as medians (Mdn) and interquartile ranges (IQRs) for all groups studied. N is the number of hearts.

		$\tau_{\lambda(alt)}$		$y_{0\lambda(alt)}$		$\lambda'_{0crit(alt)}$		$m_{max\lambda(alt)}$		$BCL_{crit\lambda(alt)}$ (ms)	
	**N**	**Mdn**	**IQR**	**Mdn**	**IQR**	**Mdn**	**IQR**	**Mdn**	**IQR**	**Mdn**	**IQR**
WT LV epicardium	20	0.55	0.78	0.01	0.03	1.47	0.59	-6.50	15.12	62.60	37.40
WT RV epicardium	21	0.66	0.98	0.01	0.01	1.04	2.81	-0.71	4.66	43.83	35.49
WT LV endocardium	7	0.88	1.39	0.05	0.05	1.43	1.57	-1.94	875.48	54.63	14.82
WT RV endocardium	8	1.49	1.97	0.05	0.10	1.67	6.21	-0.59	6.34	57.90	23.23
WT LV epicardium + flecainide	6	0.28	0.30	0.01	0.06	1.50	0.67	-5.11	210.78	80.62	43.91
WT RV epicardium + flecainide	6	0.41	0.20	0.00	0.00	0.54	1.01	-5.07	10.91	41.43	65.29
WT LV endocardium + flecainide	10	0.96	1.22	0.08	0.11	1.63	3.26	-2.47	209.63	61.46	20.68
WT RV endocardium + flecainide	8	0.53	1.51	0.04	0.06	1.13	1.13	-7.87	51.23	55.55	25.67
WT LV epicardium + quinidine	8	0.41	0.38	0.04	0.03	1.15	1.12	-5.33	540.14	63.34	44.79
WT RV epicardium + quinidine	6	1.27	1.61	0.03	0.02	0.64	3.11	-0.43	0.69	42.12	36.01
WT LV endocardium + quinidine	5	0.87	0.84	0.04	0.01	0.26	1.23	-0.98	1.31	45.63	84.80
WT RV endocardium + quinidine	5	0.27	0.04	0.09	0.11	1.13	1.83	-68.36	330.42	73.71	14.90
Scn5a$^{+/-}$ LV epicardium	13	0.64	0.28	0.01	0.01	1.52	1.23	-5.11	12.74	57.86	15.34
Scn5a$^{+/-}$ RV epicardium	24	0.41	0.28	0.00	0.01	1.23	1.12	-1.46	35.82	57.80	42.27
Scn5a$^{+/-}$ LV endocardium	6	1.78	2.54	-0.02	0.09	-1.77	6.41	-0.28	0.93	7.54	10.47
Scn5a$^{+/-}$ RV endocardium	7	0.88	0.63	0.03	0.07	0.14	1.95	-0.70	58.67	26.45	35.25
Scn5a$^{+/-}$ LV epicardium + flecainide	7	0.43	1.53	0.01	0.01	1.14	5.37	-0.54	59.08	69.09	39.47
Scn5a$^{+/-}$ RV epicardium + flecainide	8	0.29	0.40	0.01	0.02	1.00	1.29	-26.38	1888.07	97.87	8.19
Scn5a$^{+/-}$ LV endocardium + flecainide	5	1.26	0.79	0.02	0.02	1.51	1.58	-1.12	0.76	64.53	8.72
Scn5a$^{+/-}$ RV endocardium + flecainide	5	0.79	1.39	0.08	0.10	1.45	0.34	-1.24	3.08	64.74	23.74
Scn5a$^{+/-}$ LV epicardium + quinidine	6	0.39	0.35	0.04	0.08	1.10	1.09	-8.20	66.26	67.76	17.73
Scn5a$^{+/-}$ RV epicardium + quinidine	9	0.54	0.55	0.02	0.04	1.17	1.60	-3.81	119.46	96.40	3.17
Scn5a$^{+/-}$ LV endocardium + quinidine	5	0.26	0.20	0.02	0.03	1.59	0.43	-409.68	4840.20	72.47	14.45
Scn5a$^{+/-}$ RV endocardium + quinidine	5	0.94	0.69	0.02	0.04	1.51	1.06	-4.95	16.34	58.32	38.22

Table 4 – Results from comparison of λ' restitution and alternans plots. N is the number of hearts.

		q		***s***		***λ'$_{0\chi}$***		***$BCL_{\chi(\lambda)}$* (ms)**	
	**N**	**Mdn**	**IQR**	**Mdn**	**IQR**	**Mdn**	**IQR**	**Mdn**	**IQR**
WT LV epicardium	14	0.71	0.70	2.55	2.10	1.33	0.06	65.79	28.56
WT RV epicardium	14	0.42	1.93	3.18	5.34	1.11	0.51	48.03	15.98
WT LV endocardium	8	16.69	405875.74	8.25	8.91	1.72	0.87	51.90	12.74
WT RV endocardium	5	0.88	1.07	1.55	1.43	1.84	0.60	50.67	10.01
WT LV epicardium + flecainide	5	0.51	34.14	4.37	5.32	1.34	0.27	75.88	15.23
WT RV epicardium + flecainide	7	0.11	0.26	4.04	2.46	0.87	0.32	74.35	29.49
WT LV endocardium + flecainide	9	0.23	40597.12	2.59	16.65	2.55	1.06	66.57	35.38
WT RV endocardium + flecainide	7	0.18	0.36	4.02	3.09	1.41	0.68	57.30	25.66
WT LV epicardium + quinidine	9	0.60	752.59	5.23	8.88	0.99	0.37	72.23	23.03
WT RV epicardium + quinidine	7	0.71	0.64	2.60	2.23	1.27	0.29	55.88	9.40
WT LV endocardium + quinidine	6	1.05	3.98	2.15	0.45	1.57	0.12	69.93	15.23
WT RV endocardium + quinidine	6	0.02	7.57	6.36	6.25	1.11	1.37	69.98	10.06
$Scn5a^{+/-}$ LV epicardium	13	0.44	0.85	3.31	2.09	1.30	0.54	49.73	6.37
$Scn5a^{+/-}$ RV epicardium	22	0.32	3.75	3.47	4.08	1.04	0.49	53.11	22.25
$Scn5a^{+/-}$ LV endocardium	7	0.30	0.72	0.99	1.06	1.84	0.85	53.49	13.51
$Scn5a^{+/-}$ RV endocardium	7	0.10	0.26	1.67	8.03	1.31	0.39	48.60	25.81
$Scn5a^{+/-}$ LV epicardium + flecainide	6	0.02	0.16	2.44	2.15	1.42	1.11	89.44	51.36
$Scn5a^{+/-}$ RV epicardium + flecainide	7	0.11	0.21	1.33	3.25	1.01	0.16	83.28	19.97
$Scn5a^{+/-}$ LV endocardium + flecainide	5	0.34	0.48	0.98	0.52	1.57	2.11	63.27	10.19
$Scn5a^{+/-}$ RV endocardium + flecainide	5	1.62	37.62	1.95	1.86	1.30	0.82	52.28	32.55
$Scn5a^{+/-}$ LV epicardium + quinidine	6	0.15	0.26	5.13	9.95	1.14	0.98	54.26	29.96
$Scn5a^{+/-}$ RV epicardium + quinidine	7	0.11	0.16	1.37	3.20	1.04	0.41	77.82	34.43
$Scn5a^{+/-}$ LV endocardium + quinidine	5	0.00	5.19	4.64	3.14	1.59	0.48	68.28	16.63
$Scn5a^{+/-}$ RV endocardium + quinidine	6	0.17	0.25	2.01	0.69	1.87	1.40	51.25	41.71

Supplementary Table 5 – Fitting parameters that were not used in physiological analysis. Presented for use in future modeling studies.

	A_θ (ms^{-1})		$y0\theta$ (ms^{-1})		A_λ		$y_{0\lambda}$		$A_{\lambda(alt)}$	
	**Mdn**	**IQR**	**Mdn**	**IQR**	**Mdn**	**IQR**	**Mdn**	**IQR**	**Mdn**	**IQR**
WT LV epicardium	0.14	0.67	-0.06	0.63	5.64	3.32	-1.32	3.17	8.02	50.42
WT RV epicardium	0.16	0.52	-0.08	0.43	3.39	1.4	-0.99	1.44	11.4	55409.12
WT LV endocardium	2.16	16.91	-2.09	16.93	9.36	13.6	-3.84	5.87	13.23	248.26
WT RV endocardium	0.15	0.46	-0.05	0.49	5.75	2.56	-0.91	1.88	4.56	46959.57
WT LV epicardium + flecainide	0.18	0.16	-0.04	0.13	7.1	5.24	-1.33	5.28	94.65	302.42
WT RV epicardium + flecainide	9.78	72723.06	-9.74	72723.09	4.29	3.79	-0.98	3.6	3.36	6.98
WT LV endocardium + flecainide	0.24	1.94	-0.16	2	5.61	10.76	-1.66	2.87	44.09	641.9
WT RV endocardium + flecainide	0.99	36.98	-0.91	37	4.35	9.52	-1.91	5.95	8.06	161
WT LV epicardium + quinidine	0.19	115.75	-0.09	115.76	3.08	2.23	-0.28	1.69	10.78	689.04
WT RV epicardium + quinidine	0.39	0.37	-0.32	0.35	4.15	1.51	-1.02	0.66	2.18	1.93
WT LV endocardium + quinidine	0.13	0.19	-0.05	0.12	4.1	4.5	-0.57	1.73	1.52	12.79
WT RV endocardium + quinidine	0.47	4.31	-0.42	4.33	4.95	15.19	-2.69	15.14	3139.31	8847.17
Scn5a$^{+/-}$ LV epicardium	0.18	0.17	-0.13	0.17	6.05	1.66	-1.94	1.38	8.71	26.25
Scn5a$^{+/-}$ RV epicardium	0.11	1.38	-0.07	1.38	4.04	3.86	-0.97	4.98	12.78	61.38
Scn5a$^{+/-}$ LV endocardium	0.08	0.57	-0.03	0.56	7.31	267.19	-4.27	268.15	0.85	1.92
Scn5a$^{+/-}$ RV endocardium	0.15	1.26	-0.1	1.23	8.99	6.91	-3.7	23.68	1.08	49.19
Scn5a$^{+/-}$ LV epicardium + flecainide	1.92	23.69	-1.87	23.76	7.57	15.9	-4.74	14.86	6.74	264.25
Scn5a$^{+/-}$ RV epicardium + flecainide	9.26	648.07	-9.23	648.08	9.05	12.21	-7.6	12.48	33.03	5780.38
Scn5a$^{+/-}$ LV endocardium + flecainide	7.08	454.88	-3.88	6.72	13.06	10.65	-8.98	14.18	4.12	4.93
Scn5a$^{+/-}$ RV endocardium + flecainide	1.33	17.11	-1.27	17.07	6.01	10.68	-1.72	6.83	7.82	7.59
Scn5a$^{+/-}$ LV epicardium + quinidine	0.2	38.56	-0.15	38.56	4.03	3.97	-1.25	2.54	10.23	131.61
Scn5a$^{+/-}$ RV epicardium + quinidine	0.65	1.57	-0.58	1.56	4.72	25.38	-1.83	26.17	10.34	218.22
Scn5a$^{+/-}$ LV endocardium + quinidine	1.56	1.51	-1.51	1.5	456.01	1021.76	-453.29	1024.77	922.81	3346.14
Scn5a$^{+/-}$ RV endocardium + quinidine	2.33	65.01	-2.28	65.01	5.62	9.41	-1.61	5.42	8.79	9.29

Supplementary data 6 - Significant Kruskal-Wallis Tests

Kruskal-Wallis tests, which preceded detailed Bonferroni-Holm corrected Mann-Whitney U-tests. Kruskal-Wallis tests were applied to groups of conditions as detailed in the methods section. Below is a list of their significances with their associated chi-squared value (χ^2), degrees of freedom (d.f.) and P-value (P). Physiologically significant negative results are displayed in the main text.

Latency at refractoriness
WT (χ^2=47.1, d.f.=11, P=2.1x10^{-6}), *Scn5a*$^{+/-}$ (χ^2=36.9, d.f.=11, P=1.2x10^{-4}), untreated (χ^2=23.9, d.f.=7, P=0.001), flecainide-treated (χ^2=22.0, d.f.=7, P=0.003), LV epi- (χ^2=22.8, d.f.=5, P=3.7x10^{-4}) and endocardial (χ^2=13.3, d.f.=5, P=0.021), and RV epi- (χ^2=43.4, d.f.=5, P=3.1x10^{-8}) and endocardial (χ^2=13.6, d.f.=5, P=0.018) groups.

τ_θ
WT (χ^2=21.3, d.f.=11, P=0.031), flecainide-treated (χ^2=15.3, d.f.=7, P=0.032), LV epi- (χ^2=12.1, d.f.=5, P=0.032) and endocardial (χ^2=14.9, d.f.=5, P=0.011) groups

θ'_{max}
WT (χ^2=31.1, d.f.=11, P=0.001), *Scn5a*$^{+/-}$ (χ^2=25.0, d.f.=11, P=0.009), untreated (χ^2=20.2, d.f.=7, P=0.005), flecainide-treated (χ^2=21.6, d.f.=7, P=0.003), quinidine-treated (χ^2=16.4, d.f.=7, P=0.022), LV epicardial (χ^2=16.1, d.f.=5, P=0.007), and RV epi- (χ^2=26.8, d.f.=5, P=6.3x10^{-5}) and endocardial (χ^2=15.2, d.f.=5, P=0.010) groups.

$DI_{limit(\theta)}$
WT (χ^2=20.4, d.f.=11, P=0.040), *Scn5a*$^{+/-}$ (χ^2=36.5, d.f.=11, P=1.4x10^{-4}), RV epi- (χ^2=22.2, d.f.=5, P=4.8x10^{-4}) and endocardial (χ^2=17.0, d.f.=5, P=0.004) groups

Magnitude of θ' alternans at 13.5 Hz
Untreated (χ^2=20.9, d.f.=7, P=0.004) and RV epicardial (χ^2=11.2, d.f.=5, P=0.048) groups.

τ_λ
WT (χ^2=20.6, d.f.=11, P=0.038), flecainide-treated (χ^2=14.3, d.f.=7, P=0.047), quinidine-treated (χ^2=15.0, d.f.=7, P=0.036) and RV epicardial (χ^2=20.5, d.f.=5, P=0.001) groups

λ'_{max}
WT (χ^2=24.8, d.f.=11, P=0.010), *Scn5a*$^{+/-}$ (χ^2=21.4, d.f.=11, P=0.030), untreated (χ^2=30.9, d.f.=7, P=6.5x10^{-5}), RV epicardial (χ^2=15.5, d.f.=5, P=0.009) and LV endocardial (χ^2=15.3, d.f.=5, P=0.009) groups

$m_{max(\lambda)}$
Quinidine-treated (χ^2=20.8, d.f.=7, P=0.004) and RV epicardial (χ^2=17.8, d.f.=5, P=0.004) groups

λ'_{0limit}
WT (χ^2=22.4, d.f.=11, P=0.021), *Scn5a*$^{+/-}$(χ^2=25.7, d.f.=11, P=0.007), flecainide-treated (χ^2=14.6, d.f.=7, P=0.041) and quinidine-treated (χ^2=15.7, d.f.=7, P=0.028) groups.

$BCL_{crit(\lambda)}$
Scn5a$^{+/-}$ (χ^2=26.5, d.f.=11, P=0.005), RV epi- (χ^2=21.6, d.f.=5, P=0.001) and endocardial (χ^2=12.1, d.f.=5, P=0.034) groups.

BCD' at 8 Hz

WT (χ^2=44.0, d.f.=11, P=7.4x10^{-6}), untreated (χ^2=29.4, d.f.=7, P=1.2x10^{-4}), flecainide (χ^2=22.4, d.f.=7, P=0.002), LV epi- (χ^2=20.2, d.f.=5, P=0.001) and endocardial (χ^2=13.8, d.f.=5, P=0.017), and RV epi- (χ^2=31.3, d.f.=5, P=8.1x10^{-6}) and endocardial (χ^2=13.2, d.f.=5, P=0.0.022) groups.

BCD' at 13.5 Hz
WT (χ^2=27.7, d.f.=11, P=0.004), *Scn5a*$^{+/-}$ (χ^2=33.9, d.f.=11, P=3.7x10^{-4}), untreated (χ^2=21.0, d.f.=11, P=0.004), flecainide-treated (χ^2=14.4, d.f.=7, P=0.044), quinidine-treated (χ^2=18.5, d.f.=7, P=0.010), RV epi- (χ^2=15.9, d.f.=5, P=0.007) and endocardial (χ^2=26.147, d.f.=5, P=8.4x10^{-5}) groups.

$\tau_{\lambda(alt)}$
WT (χ^2=19.9, d.f.=11, P=0.046), *Scn5a*$^{+/-}$(χ^2=21.6, d.f.=11, P=0.028), untreated (χ^2=16.0, d.f.=7, P=0.025) and quinidine-treated (χ^2=15.5, d.f.=7, P=0.030) groups.

$y_{0\lambda(alt)}$
WT (χ^2=29.1, d.f.=11, P=0.002), untreated (χ^2=17.4, d.f.=7, P=0.015) and LV endocardial (χ^2=11.3, d.f.=5, P=0.046) groups.

$BCL_{crit\lambda(alt)}$
Scn5a$^{+/-}$ (χ^2=30.9, d.f.=11, P=0.001), untreated (χ^2=15.5, d.f.=7, P=0.030), flecainide (χ^2=14.7, d.f.=7, P=0.039) and RV epicardial (χ^2=21.6, d.f.=5, P=0.001) groups.

$\lambda'_{0\chi}$
WT (χ^2=32.2, d.f.=11, P=0.001), untreated (χ^2=17.7, d.f.=7, P=0.013) and flecainide groups (χ^2=17.6, d.f.=7, P=0.014).

$BCL_{\lambda(\chi)}$
WT (χ^2=20.4, d.f.=11, P=0.040) and RV epicardial (χ^2=12.9, d.f.=5, P=0.025) groups.

Supplementary data 7 – Additional Mann-Whitney Significances

Latency at refractoriness

Conduction velocities from the latencies of the AP records at the BCL just preceding refractoriness. Flecainide additionally increased the latencies in the WT LV epicardium and RV epicardium (from 18.43(13.35) to 40.31(13.34) ms; U=36.0, P=0.017, r=0.47 and from 18.31(6.99) to 49.34(9.79) ms; U=10.0, P=0.004, r=0.58 respectively)..

Quinidine increased conduction times in all WT recording sites (LV epicardium: from 18.43(13.35) to 39.94(12.23) ms; U=33.0, P=0.002, r=0.57; RV epicardium: from 18.31(6.99) to 25.89(8.88) ms; U=49.0, P=0.013, r=0.48; LV endocardium: from 21.06(11.77) to 36.91(12.93) ms; U=11.0, P=0.013, r=0.59 and RV endocardium: from 19.01(16.46) to 41.41(12.89) ms; U=16.0, P=0.018, r=0.57). However, in the *Scn5a*$^{+/-}$, quinidine additionally increased conduction latency in the RV endocardium only (from 16.74(9.20) to 26.19(21.21) ms; U=18.0, P=0.037, r=0.57). Unlike flecainide, quinidine decreases θ in both the WT and

Scn5a$^{+/-}$. As decreased θ is associated with arrhythmogenicity, this potentially limits the therapeutic use of quinidine.

Together these findings demonstrated that before pharmacological intervention, the *Scn5a*$^{+/-}$ RV epicardium shows a slowed conduction likely reflecting a right sided conduction block. This results in a transmural difference in conduction times not present in the WT. Either flecainide or quinidine slowed conduction velocity in both genotypes as expected from their Na^+ channel blocking activity.

τ_λ

As with the λ' against DI plots, a time constant τ_λ could be used to describe the width of the λ' restitution curve, which was only altered by quinidine. Quinidine decreased τ_λ in the WT RV endocardium (from 4.07(3.26) to 1.26(1.39); U=3.0, P=0.027, r=0.72). In the presence of quinidine, τ_λ was smaller in the WT RV endocardium (1.26(1.39)), than in the corresponding epicardium (2.74(2.22); U=4.0, P=0.006, r=0.75). Thus, as in the previous case, τ_λ was not predictive of arrhythmic tendency.

BCD' at 8Hz

The basic cycle distance BCD, given by BCD' = BCL/latency determines the location on the λ restitution curve at any given pacing rate (see Theory section). Flecainide reduced BCD' in most WT areas. (WT LV epicardium: from 9.49(6.43) to 4.67(3.05); U=46.0, P=0.003, r=0.51; WT RV epicardium: from 10.28(10.09) to 4.55(2.91); U=24.5, P=0.002, r=0.59; WT RV endocardium: from 13.8(12.67) to 8.25(5.30); U=17.0, P=0.048, r=0.50). BCD' in the flecainide-treated WT LV epicardium (4.67(3.05) was smaller than in corresponding endocardium (9.06(3.98); U=15.0, P=0.005, r=0.66). Quinidine decreased BCD' in WT LV epicardium and (from 9.49(6.43) to 6.29(5.08); U=78.0, P=0.013, r=0.44). These differences reflect flecainide's and quindine's Na^+ channel blocking actions which slow conduction velocity.

BCD' at 13.5Hz

At an exercising heart rate of 13.5 Hz (Figure 7C), BCD' in flecainide reduced the BCD' at 13.5Hz in the WT RV endocardium (from 8.94(8.55) to 4.17(1.01); U=5.0, P=0.038, r=0.70). Quinidine exerted marked actions particularly in the WT RV endocardium. The BCD' at 13.5Hz in the quinidine-treated WT RV endocardium was significantly smaller than in the corresponding epicardium (3.00(1.94) vs. 5.48(3.94); U=0.0, P=0.014, r=0.81). Finally, BCD' in the quinidine-treated WT RV endocardium 1.09(1.06) was smaller than in the corresponding flecainide-treated area (4.17(1.01); U=3.0, P=0.025, r=0.73). Additionally,

quinidine reduced BCD' in the *Scn5a*$^{+/-}$ LV epicardium and the *Scn5a*$^{+/-}$ RV endocardium ((from 5.76(3.94) to 4.14(1.13); U=10.0, P=0.015, r=0.63) and from 5.27(1.88) to 3.00(1.94); U=6.0, P=0.009, r=0.70 respectively). Thus, quinidine results in a reduced BCD' in the WT RV endocardium, as compared to the *Scn5a*$^{+/-}$, WT RV epicardium or the action of flecainide. This implicates the WT RV endocardium as the site for arrhythmia initiation in the presence of quinidine. Quinidine also reduces BCD' in the *Scn5a*$^{+/-}$ which may result in pro-arrhythmic effects potentially limiting its use in therapy.

$\tau_{\lambda(alt)}$

The curvature of the λ' alternans plots, $\tau_{\lambda(alt)}$ was decreased by quinidine in the WT RV endocardium (from 1.49(1.97) to 0.27(0.04); U=0.0, P=0.014, r=0.81). $\tau_{\lambda(alt)}$ was greater in the quinidine-treated WT RV epicardium than in the WT RV endocardium (1.27(1.61) vs. 0.27(0.04); U=0.0, P=0.025, r=0.82). Quinidine also decreased the value of $\tau_{\lambda(alt)}$ in the *Scn5a*$^{+/-}$ LV endocardium (from 1.78(2.54) to 0.26(0.20); U=1.0, P=0.042, r=0.77). Again in keeping with the λ' restitution curves, these results demonstrate that quinidine is able to alter the broadness of the function.

$m_{max(\lambda)}$

The maximum gradient, $m_{max(\lambda)}$, of the λ' restitution curve typically took a value of 1.7(3.45), exceeding a value of one., with the exception of the quinidine-treated WT RV epicardium compared to the corresponding *Scn5a*$^{+/-}$ (1.03(0.51) vs. 2.68(9.79); U=13.0, P=0.017, r=0.66) and the quinidine-treated WT LV endocardium compared to the corresponding *Scn5a*$^{+/-}$ (0.97(1.34) vs. 18.30(81.13); U=16.0, P=0.031, r=0.09).
