# Supplementary material for: Action potential wavelength restitution predicts alternans and arrhythmia in murine Scn5a+/− hearts
Source: J Physiol. 2013 Jul 8;591(Pt 17):4167–88. doi: 10.1113/jphysiol.2013.254938 (PMC3779110; doi:10.1113/jphysiol.2013.254938)

Supplementary figure 1: Conduction velocity restitution curves plotting θ' against DI obtained from typical (N=1) WT (open symbols; black lines) and *Scn5a*[+/-] (filled symbols; grey lines) hearts in the (A) LV epicardium and (B) LV endocardium, before (circles; solid line) and following addition of flecainide (squares; dashed line) or quinidine (triangles; dotted line). The data displayed represents hearts showing the Mdn value of θ'_{max} in the experimental group concerned.

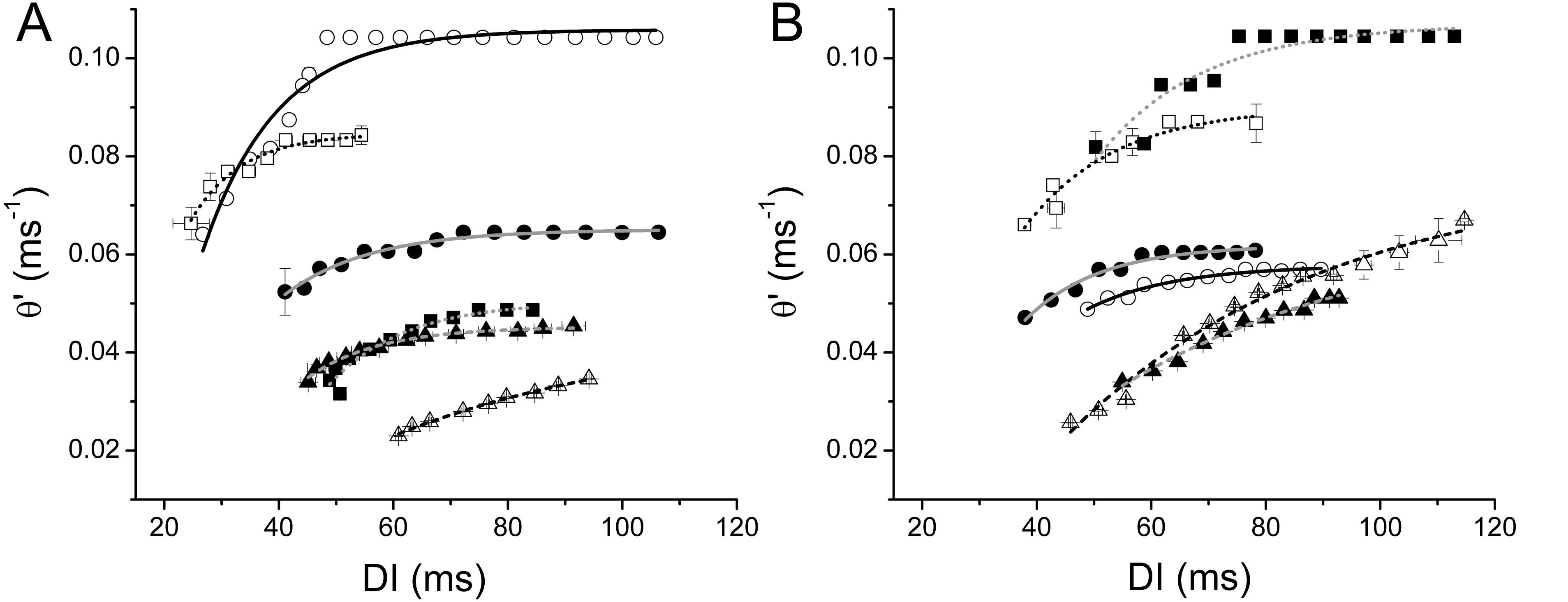

Supplementary Figure 2: Box and whisker plots summarizing the magnitude of θ’ alternans in the LV at resting (8 Hz) (white plots) and exercising (13.5 Hz) (filled plots) heart rates. Horizontal lines denote the Mdn, the box denotes the IQR and the whisker the range. No significant differences were observed.

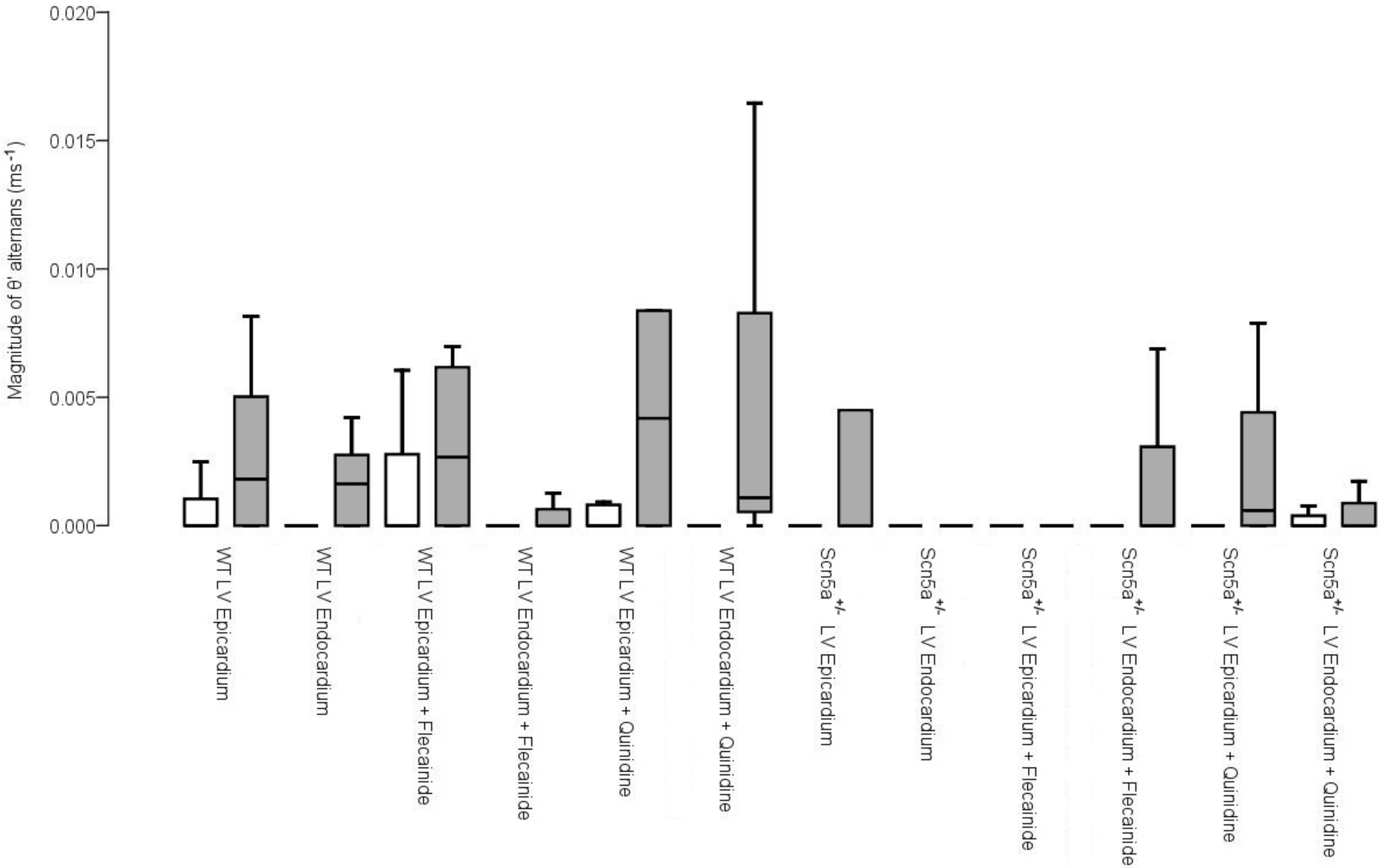

Supplementary Figure 3: Plots of λ' against DI obtained from the typical (N=1) WT (open symbols; black lines) and *Scn5a*$^{+/-}$ (closed symbols; grey lines) hearts in the (A) LV epicardium and (B) LV endocardium, before (circles; solid line) and following addition of flecainide (squares; dashed line) or quinidine (triangles; dotted line). The data displayed represents hearts showing the Mdn value of λ'_{max} in the experimental group concerned.

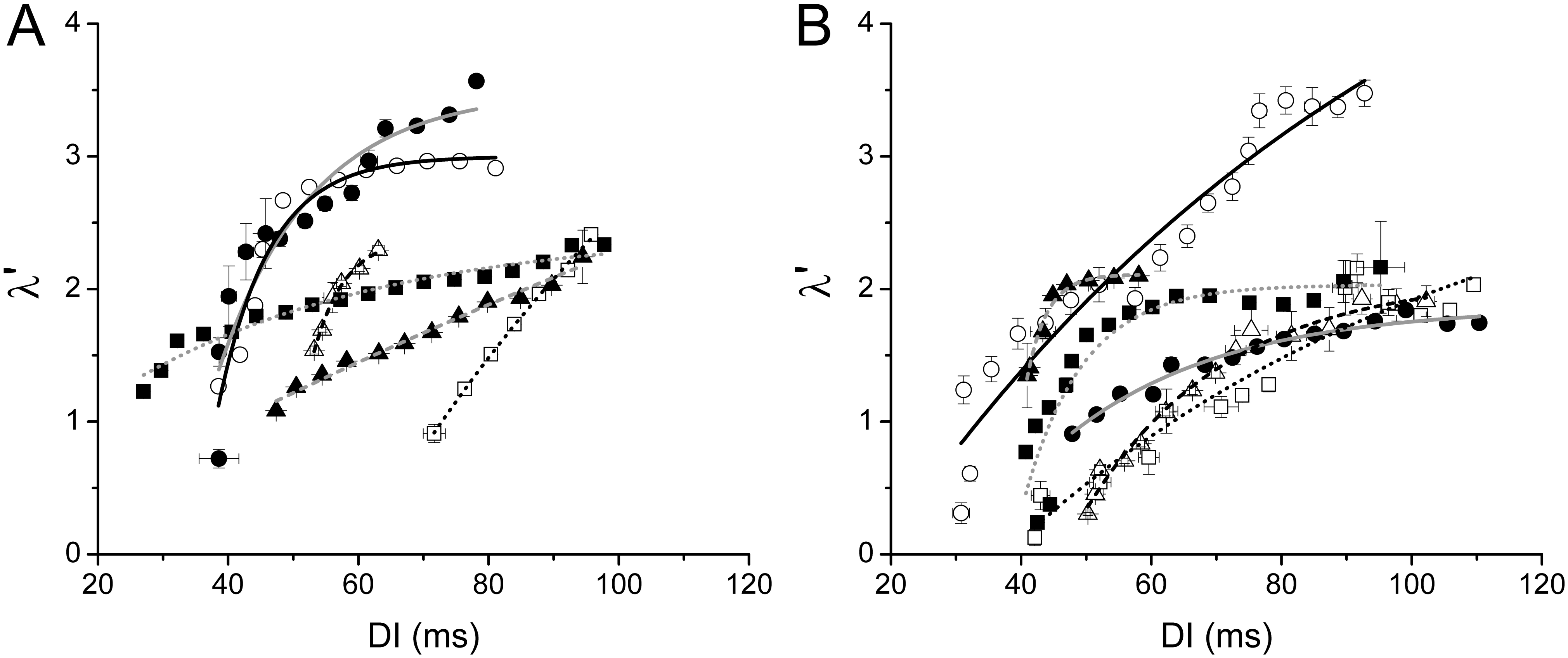

Supplementary Figure 4: λ' restitution curves plotting λ' against λ'_0 obtained from the typical (N=1) WT (open symbols; black lines) and *Scn5a*$^{+/-}$ (closed symbols; grey lines) hearts in the (A) LV epicardium and (B) LV endocardium, before (circles; solid line) and following addition of flecainide (squares; dashed line) or quinidine (triangles; dotted line). The data displayed represents hearts showing the Mdn value of λ'_{max} in the experimental group concerned.

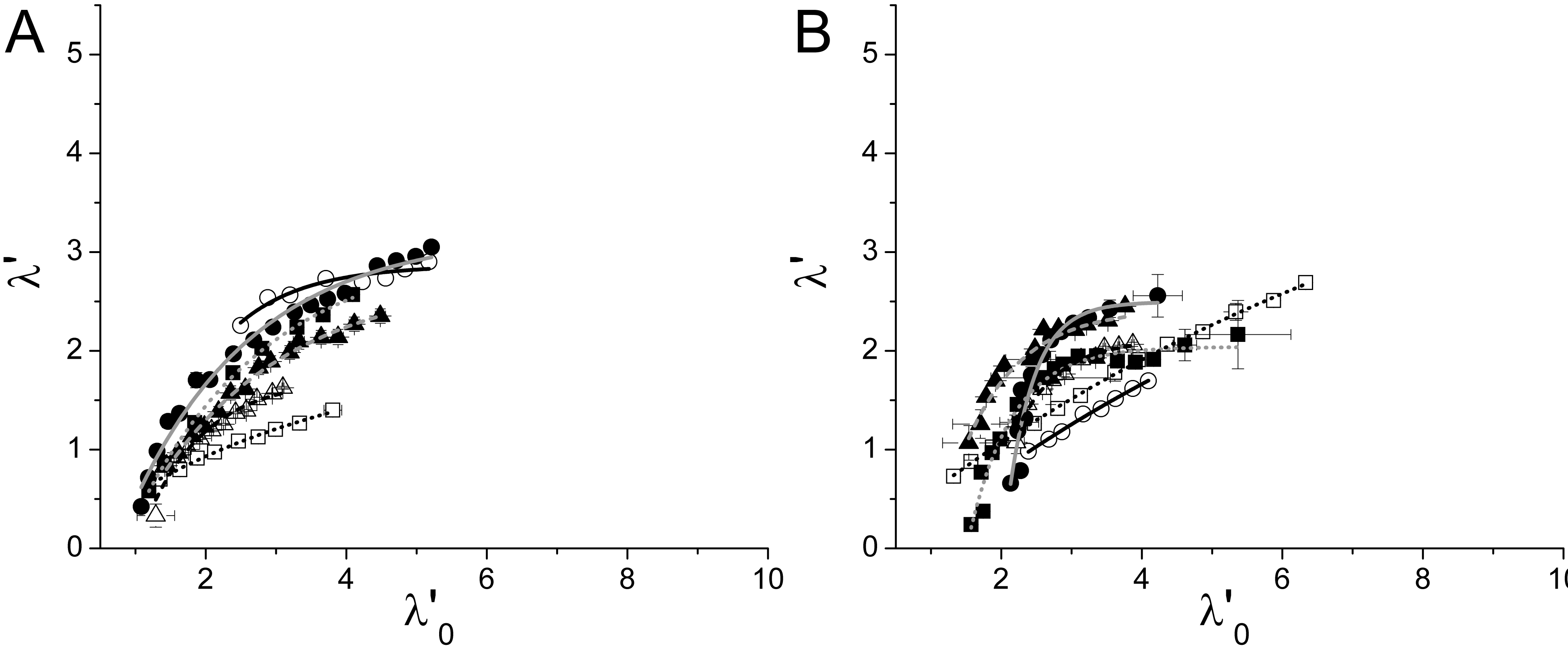

Supplementary Figure 5: Changes in the BCD' shown in box and whiskers plots at 8 Hz (white plots) and 13.5Hz (filled plots) pacing in the LV. Horizontal lines indicate the Mdn, boxes the IQR and whiskers show the range. * Denotes significant differences P<0.05, solid lines show comparisons between 8Hz pacing and dashed lines show comparisons between 13.5Hz pacing.

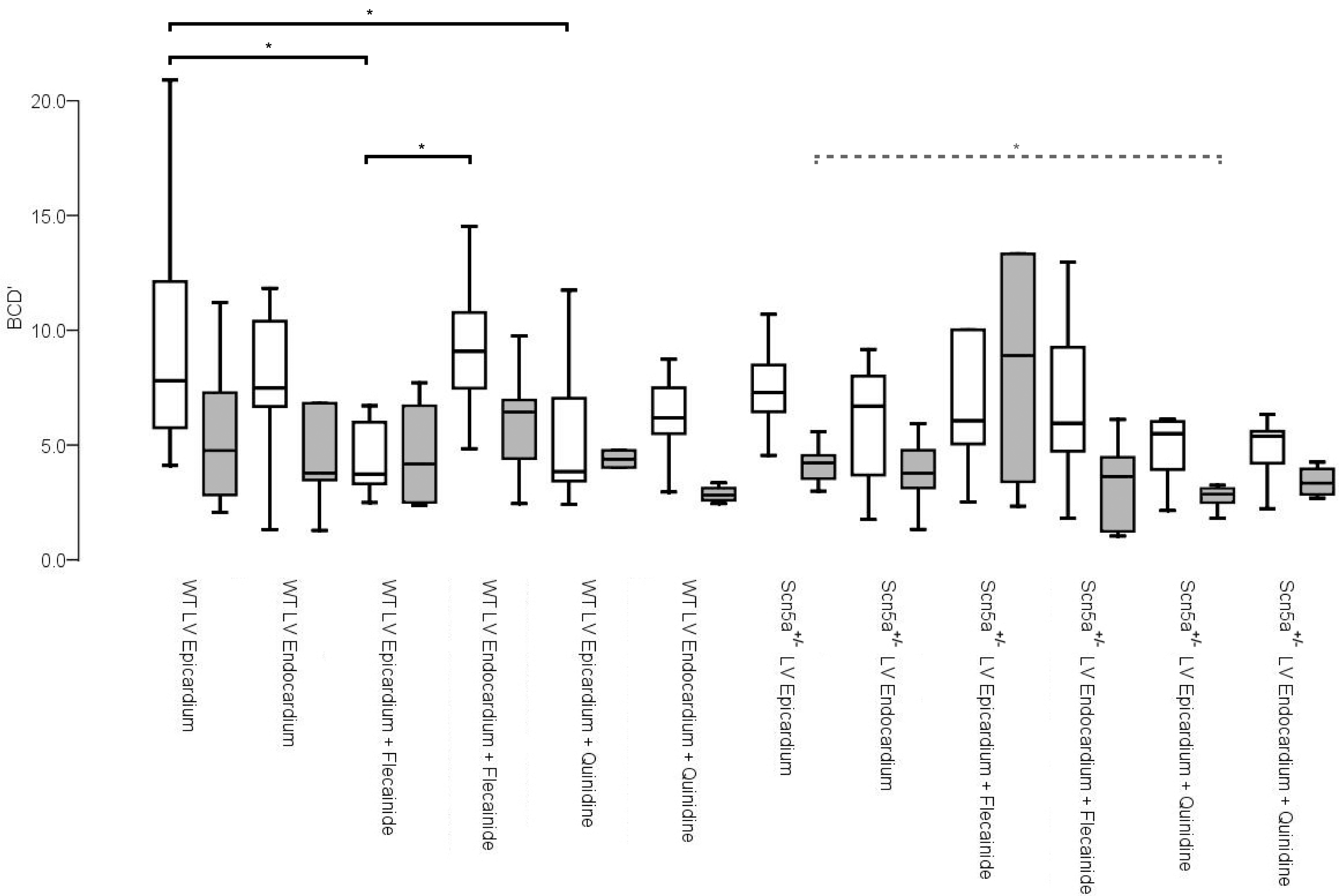

Supplementary Figure 6: Typical (N=1) plots of the magnitude of λ' alternans, $mag_{\lambda\,(alt)}$ against λ'_0 in WT (open symbols; black lines) and *Scn5a*$^{+/-}$ (closed symbols; grey lines), (A) LV epicardium, and (B) LV endocardium, before (circles; solid line) and following addition of either flecainide (squares; dashed lines) or quinidine (triangles; dotted lines). Data represents the Mdn value of $\lambda'_{0crit(alt)}$ as all curves progress together with common negative gradients.

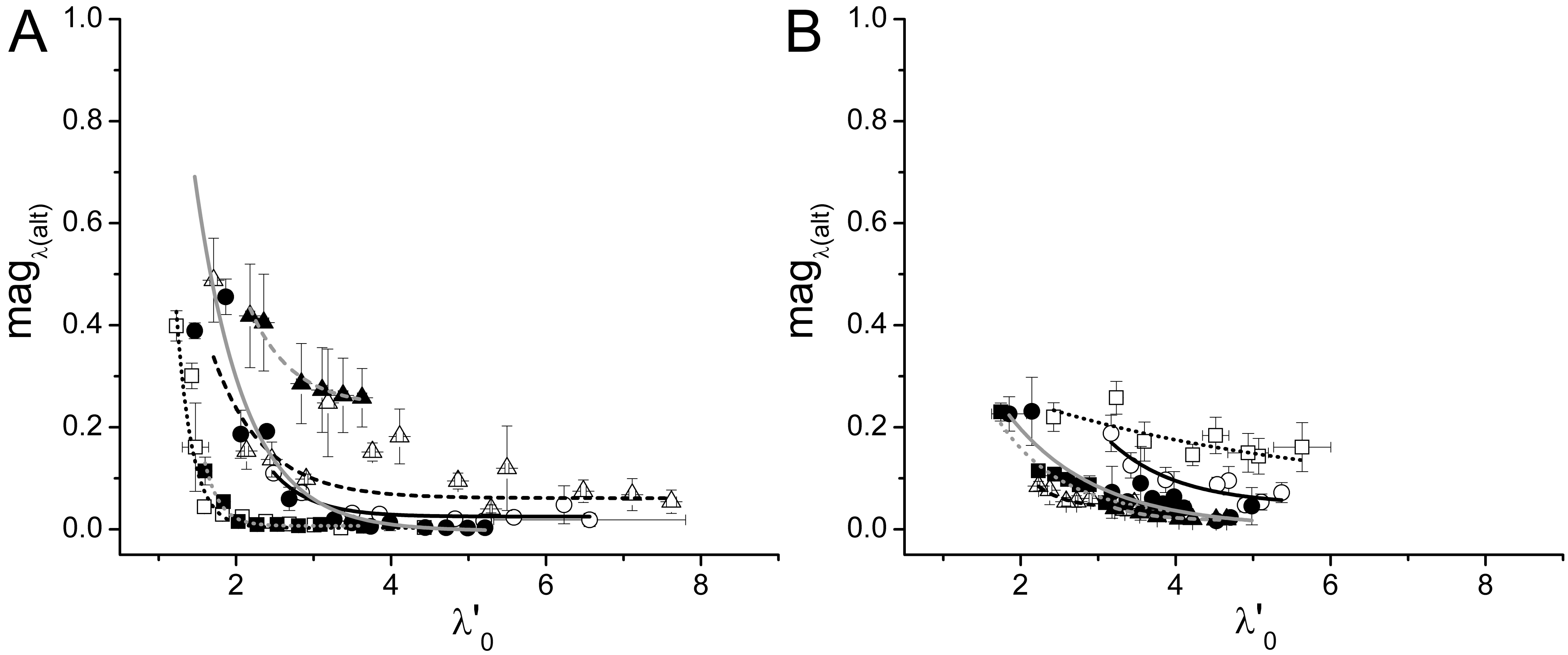

Supplement: Supplementary file 2 [file tjp0591-4167-SD2.pdf]
